# Transdiagnostic Neurocognitive Endophenotypes for Schizophrenia, Bipolar I Disorder and a Broad Psychosis/Bipolar I Disorder Phenotype: A Mega-Analysis of Twin and Sibling Data

**DOI:** 10.1093/schbul/sbaf050

**Published:** 2025-05-09

**Authors:** Eugenia Kravariti, Anna-Maria Fragkaki, Anna Georgiades, Alastair G Cardno, Fergus Kane, Sridevi Kalidindi, Katja K Schulze, Colm McDonald, Marco M Picchioni, Mei-Hua Hall, Cameron J Watson, Birte Y Glenthøj, Bjørn H Ebdrup, Birgitte Fagerlund, Cecilie K Lemvigh, Neeltje E M Van Haren, Rene Kahn, Robin M Murray, Fruhling Rijsdijk, Timothea Toulopoulou

**Affiliations:** Department of Psychosis Studies, Institute of Psychiatry, Psychology, and Neuroscience, King’s College London, London SE5 8AF, United Kingdom; Department of Psychosis Studies, Institute of Psychiatry, Psychology, and Neuroscience, King’s College London, London SE5 8AF, United Kingdom; First Department of Psychiatry, School of Medicine, National and Kapodistrian University of Athens 115 27, Greece, Athens, Greece; Department of Psychosis Studies, Institute of Psychiatry, Psychology, and Neuroscience, King’s College London, London SE5 8AF, United Kingdom; Brent Early Intervention Service, CNWL, NHS Foundation Trust, 27-29 Fairlight Avenue, London NW10 8AL, United Kingdom; Division of Psychological and Social Medicine, Faculty of Medicine and Health, University of Leeds, Leeds LS2 9JT, United Kingdom; Department of Clinical, Educational and Health Psychology, University College London, London WC1E 6BT, United Kingdom; Recovery and Rehabilitation Team, Croydon Directorate, South London and Maudsley NHS Foundation Trust, London CR0 2PR, United Kingdom; Centre for Anxiety Disorders and Trauma (CADAT), South London and Maudsley NHS Foundation Trust, London SE5 8AZ, United Kingdom; Centre for Neuroimaging & Cognitive Genomics (NICOG), Clinical Neuroimaging Laboratory, Galway Neuroscience Centre, College of Medicine Nursing and Health Sciences, University of Galway, Galway H91 TK33, Ireland; Department of Forensic and Neurodevelopmental Science, Institute of Psychiatry, Psychology and Neuroscience, King’s College London, London SE5 8AF, United Kingdom; Psychosis Neurobiology Laboratory, McLean Hospital, Harvard Medical School, Belmont, Massachusetts MA 02478, United States; South London and Maudsley NHS Foundation Trust, London SE5 8AZ, United Kingdom; Neuropsychiatry Research and Education Group Institute of Psychiatry, Psychology and Neuroscience, King’s College London, London SE5 8AF, United Kingdom; Social, Genetic and Developmental Psychiatry Centre, Institute of Psychiatry, Psychology and Neuroscience, King’s College London SE5 8AF, London, United Kingdom; Center for Neuropsychiatric Schizophrenia Research (CNSR)/Center for Clinical Intervention and Neuropsychiatric Schizophrenia Research (CINS), Mental Health Center Glostrup, DK 2600, Copenhagen University Hospital – Mental Health Services CPH, Copenhagen, Denmark; Department of Clinical Medicine, Faculty of Health and Medical Sciences, University of Copenhagen, 2200 Copenhagen, Denmark; Center for Neuropsychiatric Schizophrenia Research (CNSR)/Center for Clinical Intervention and Neuropsychiatric Schizophrenia Research (CINS), Mental Health Center Glostrup, DK 2600, Copenhagen University Hospital – Mental Health Services CPH, Copenhagen, Denmark; Department of Clinical Medicine, Faculty of Health and Medical Sciences, University of Copenhagen, 2200 Copenhagen, Denmark; Center for Neuropsychiatric Schizophrenia Research (CNSR)/Center for Clinical Intervention and Neuropsychiatric Schizophrenia Research (CINS), Mental Health Center Glostrup, DK 2600, Copenhagen University Hospital – Mental Health Services CPH, Copenhagen, Denmark; Child and Adolescent Mental Health Center, Copenhagen University Hospital – Mental Health Services CPH, 2900 Hellerup, Denmark; Department of Psychology, Faculty of Social Sciences, University of Copenhagen, 1353 Copenhagen K, Denmark; Center for Neuropsychiatric Schizophrenia Research (CNSR)/Center for Clinical Intervention and Neuropsychiatric Schizophrenia Research (CINS), Mental Health Center Glostrup, DK 2600, Copenhagen University Hospital – Mental Health Services CPH, Copenhagen, Denmark; Department of Child and Adolescent Psychiatry/Psychology, Erasmus University Medical Center, 3000 CA Rotterdam, Netherlands; Department of Psychiatry, Icahn School of Medicine at Mount Sinai, Park Ave, New York, NY 10029, United States; Department of Psychosis Studies, Institute of Psychiatry, Psychology, and Neuroscience, King’s College London, London SE5 8AF, United Kingdom; Department of Psychosis Studies, Institute of Psychiatry, Psychology, and Neuroscience, King’s College London, London SE5 8AF, United Kingdom; Psychology Department, Faculty of Social Sciences, Anton de Kom University of Suriname, P.O.B. 9212 Paramaribo, Suriname, South America; First Department of Psychiatry, School of Medicine, National and Kapodistrian University of Athens 115 27, Greece, Athens, Greece; Department of Psychology & National Magnetic Resonance Research Center (UMRAM), Aysel Sabuncu Brain Research Centre (ASBAM), Bilkent University, Ankara 06800, Turkey; Department of Psychiatry, Icahn School of Medicine at Mount Sinai, Park Ave, New York, NY 10029, United States

**Keywords:** transdiagnostic, endophenotypes, schizophrenia, bipolar I disorder, twins, cognition

## Abstract

**Background:**

Psychiatric research is increasingly embracing a paradigm shift from categorical diagnoses to neurobiologically meaningful dimensions that cross current diagnostic boundaries. This transposition calls for redefining endophenotypes to accommodate transdiagnostic vulnerabilities. We sought to identify shared and disorder-specific neurocognitive endophenotypes for schizophrenia, bipolar I disorder (BD-I) and a broad psychosis/BD-I phenotype in a mega-analysis of twin/sibling data.

**Study Design:**

We performed genetic model fitting to intelligence (IQ) and computerised neurocognitive data derived from 1050 twins/siblings from three research centres in the UK, Denmark and the Netherlands, affected (*n* = 257) or unaffected (*n* = 793) by schizophrenia, other primary psychoses and BD-I. We examined the endophenotypic status of IQ, spatial working memory (SWM), visual recognition, sustained attention/rapid visual processing (RVP), mental flexibility, and spatial planning/problem solving (all validated as endophenotypes for schizophrenia in previous studies) in relation to schizophrenia, BD-I and the broad phenotype.

**Study Results:**

After covarying for age, gender, education and research centre, IQ and SWM emerged as transdiagnostic endophenotypes, showing statistically significant heritabilities (*h*^2^ 67–75% and 28–30%, respectively), phenotypic correlations (*r*_ph_ |0.14|-|0.25|) and genetic correlations (*r*_g_ |0.18|-|0.42|) with all diagnostic phenotypes. Additionally, all remaining cognitive domains received validation as endophenotypes for the broad phenotype, and all, but RVP, for schizophrenia.

**Conclusions:**

IQ and SWM tap into transdiagnostic elements of the genetic vulnerabilities to psychosis and BD-I. Our findings add to emergent evidence which spurs cautious optimism that a psychiatric nosology based on aetiology rather than phenotypical classifications may be feasible in the future, enabling biotyping and novel approaches to treatment.

## Introduction

The past decades have seen an increasing recognition that diagnostic categories of severe mental illness often fail to align with findings from clinical neuroscience, genetics and epidemiology, or fall short of predicting treatment response.^1–3^ The main reason for this misalignment is that classifications fail to co-segregate with underlying mechanisms of dysfunction.^1,2^ Such mechanisms do not respect diagnostic boundaries, and remain poorly understood, slowing the development of effective new treatments.^1,2^ In response to this recognition, psychiatric research is increasingly embracing a paradigm shift from categorical diagnoses to neurobiologically meaningful dimensions that cross diagnostic boundaries.^4,5^ This transposition relies critically on reliable biomarkers,^4,5^ including endophenotypes. The latter are heritable and measurable traits whose genetic basis is shared with and can provide clues to the genetic architecture and pathophysiological unfolding of complex psychiatric disorders.^6^

The divide and transition between diagnostic and neurobiological typologies in psychiatry are best illustrated in our evolving understanding and representation of schizophrenia (SZ) and bipolar disorder (BD).^7^ Despite being conceptualized as distinct diagnostic categories, SZ and BD show substantial genetic sharing (with genetic correlation estimates of 0.6–0.7, as indexed mainly by common variants),^8–11^ symptom cross-over, commonalities in antipsychotic response, and overlapping cognitive deficits.^12–14^ The shared genetics and neurobiology of these and other disorders are the focus of intensive research. For example, using clusters of endophenotypes relating to brain function, some studies have identified, replicated, and cross-validated a number of neurobiologically distinct psychosis profiles or “biotypes” that transcend clinical diagnosis boundaries.^5,15^ Such findings support the possibility of including laboratory measures in psychosis subtyping.

Characterizing psychiatric illness along neurobiologically meaningful dimensions may herald advances in classification, causal modeling, and treatment, but also necessitates a redefinition of the endophenotype concept to accommodate transdiagnostic vulnerabilities.^16^ Specifically, allowing for the possibility of transdiagnostic biotypes arising from transdiagnostic genetic pathways calls for a relaxation of the disorder-specificity requirement of endophenotypes.^16^

Neurocognitive measures are among the most stable and replicable candidate endophenotypes for SZ and BD.^17,18^ In SZ, genetic model-fitting analyses of twin data have given rise to formal estimates of heritability and phenotypic/genetic correlations.^19–26^ Several of these studies have endorsed IQ, episodic and working memory, attention, and executive dysfunctions as endophenotypes for SZ.^19–23,26^ By contrast, a dearth of sufficiently powered twin designs in BD has necessitated a reliance on the indirect approach of evaluating the evidence in relation to six endophenotypic criteria (association with BD, heritability, state independence, co-segregation, familial association, disorder specificity)—mostly *across* studies (i.e., each study usually evaluates just some of the criteria).^18^ This literature has highlighted attention and executive dysfunctions as promising candidate endophenotypes for BD.^18^ The only BD study to have performed comprehensive cognitive assessments and genetic model-fitting analyses of twin and sibling data has validated IQ and episodic and spatial working memory as endophenotypes for BD.^27^

To date, no twin study has used advanced genetic modeling techniques to identify transdiagnostic and disorder-specific cognitive endophenotypes for SZ, bipolar I disorder (BD-I) and a broad diagnostic phenotype that subsumes the former two categories. To address this gap in the literature, we performed a mega-analysis of IQ and CANTAB^28^ (Cambridge Neuropsychological Test Automated Battery) data using twin and sibling samples from three research centres in the UK, Denmark, and the Netherlands.

## Methods

Our study is a mega-analysis of novel, previously unpublished CANTAB^28^ data from a schizophrenia (SZ) twin series in the United Kingdom^19^ and from a twin study of bipolar disorder (BD) in the Netherlands,^29^ as well as previously published CANTAB^28^ data from a bipolar twin and sibling study in the United Kingdom^27^ and from a nation-wide twin study of schizophrenia-spectrum disorders in Denmark.^26^ Our analytic dataset further included previously unpublished IQ data from the Dutch centre^29^ and previously published IQ data from the remaining centers.^19,26,27^ The methods and procedures (sampling frame, recruitment strategy, inclusion, and exclusion criteria, assessment of zygosity and clinical characteristics, ethical approval, and consent procedures) of the individual studies have been described in detail elsewhere^19,26,27,29^ and are summarized in Supplementary Table S1.

### Participants

The zygosity, demographic, and diagnostic characteristics of the analytic cohort, divided into three diagnostic phenotypes [SZ, Bipolar I Disorder (BD-I), Broad Psychosis/BD-I phenotype; the broad phenotype included SZ, other primary psychoses, and BD-I] and an “unaffected” group (all non-psychotic, non-bipolar twins/siblings from unaffected and discordant pairs), are presented in Table 1. The analytic cohort comprised 1050 participants aged 16-65 years with IQ=>70 (after excluding 31 SZ participants with IQ < 70 from the United Kingdom and Danish samples), including 145 (108 monozygotic/MZ) twins with SZ; 75 (52 MZ) twins and 11 siblings with BD-I; 26 (16 MZ) twins with primary psychoses other than SZ (schizoaffective disorder, depressive psychosis, schizotypal disorder, brief psychotic disorder, unspecified non-organic psychosis); and 735 (438 MZ) twins/58 siblings unaffected by psychotic or bipolar disorders (Table 1). The unaffected participants were drawn from 287/24 complete control twin/sibling pairs with no personal or family histories of psychotic or bipolar disorders; from 15 incomplete control pairs; from 120/9 complete twin/sibling pairs discordant for SZ, BD-I or other primary psychoses; and from 26/1 incomplete twin/sibling pairs discordant for SZ, BD-I or other primary psychoses. A detailed narrative description of the analytic sample is included in Appendix A (Supplementary Material).

**Table 1:** Descriptive Characteristics of Participants with Schizophrenia (SZ), Bipolar I Disorder (BD-I) a Broad Psychosis/BD-I Phenotype and Those Unaffected by Psychotic/Bipolar Disorders

	Narrow phenotypes ^a^		Broad phenotype ^a^	Unaffected ^a^
	Schizophrenia (*n* = 145)	Bipolar I disorder (*n* = 86)	Psychosis/bipolar I disorder (*n* = 257)	No psychotic or bipolar disorders (*n* = 793)
**Centre, *n* (%)**				
United Kingdom—Schizophrenia Study	114 (78.62)	1 (1.16)	115 (44.75)	344 (43.38)
United Kingdom—Bipolar Disorder Study	-	75 (87.21)	79 (30.74)	247 (31.15)
Denmark—Schizophrenia Study	27 (18.62)	-	49 (19.07)	146 (18.41)
Netherlands—Bipolar Disorder Study	4 (2.76)	10 (11.63)	14 (5.45)	56 (7.06)

Concordance, *n* (%)				
From pairs concordant for the narrow phenotype	64 (44.14)	26 (30.23)	90 (35.02)	-
From pairs discordant for the narrow phenotype	66 (45.52)	45 (52.33)	111 (43.19)	111 (14.00)
From pairs concordant for the broad phenotype	4 (2.76)	7 (8.14)	16 (6.23)	-
From pairs discordant for the broad phenotype	-	-	18 (7.00)	18 (2.27)
From pairs unaffected by psychotic/bipolar disorders	-	-	-	622 (78.44)
From incomplete pairs	11 (7.59)	8 (9.30)	22 (8.56)	42 (5.30)

Zygosity/sibship, *n* (%)				
Monozygotic twin	108 (74.48)	52 (60.47)	176 (68.48)	438 (55.23)
Dizygotic twin	37 (25.52)	23 (26.74)	70 (27.24)	297 (37.45)
Sibling	-	11 (12.79)	11 (4.28)	58 (7.31)

Gender, *n* (%)				
Men	96 (66.21)	30 (34.88)	132 (51.36)	251 (31.65)
Women	49 (33.79)	56 (65.12)	125 (48.64)	542 (68.35)
**Age, years: mean (s.d.)**	36.64 (10.58)	43.31 (12.68)	39.29 (11.90)	42.01 (12.01)
**Education, years: mean (s.d.)**	13.33 (2.83)	14.36 (2.89)	13.76 (2.89)	14.54 (2.82)
**IQ: mean (s.d.)**	94.06 (14.23)	107.44 (13.01)	100.19 (14.91)	108.95 (13.74)

Primary DSM-IV diagnosis, *n* (%)				
Schizophrenia	145 (100.00)	-	145 (56.42)	-
Bipolar I disorder	-	86 (10.00)	86 (33.46)	-
Psychotic disorder other than SZ/BD-I	-	-	26 (10.12) ^b^	-
Non-psychotic, non-bipolar psychiatric disorder	-	-	-	82 (10.34) ^c^
**Recorded history of psychotic symptoms, yes: *n* (%)**	145 (100.00)	56 (65.12)	227 (88.33)	-

^a^ We performed three sets of genetic model fitting analyses: 1—“narrow schizophrenia phenotype,” 2—“narrow bipolar I disorder phenotype” and 3—‘broad psychosis/bipolar I disorder phenotype’. Unaffected participants were included in all three sets of analyses; ^b^ schizoaffective disorder (*n* = 6), depressive psychosis (*n* = 2), schizotypal personality disorder (*n* = 9), brief psychotic disorder (*n* = 8), unspecified non-organic psychosis (*n* = 1); ^c^ unipolar depressive disorders (*n* = 46), neurotic, stress-related and somatoform disorders (*n* = 15), mental and behavioral disorders due to psychoactive substance use (*n* = 7), autism-spectrum disorders (*n* = 1), disorders of adult personality and behaviour (*n* = 1), a combination of the aforementioned diagnoses (n = 12). No participant was acutely ill at the time of data collection.

Of the 86 BD-I participants, 56 (65%) had a recorded history of psychotic symptoms. Non-psychotic, non-bipolar psychiatric disorders were not an exclusion criterion for unaffected participants. Of the 793 unaffected twins and siblings, 82 had personal histories of unipolar depressive disorders (*n* = 46), neurotic, stress-related, and somatoform disorders (*n* = 15), mental and behavioural disorders due to psychoactive substance use (*n* = 7), autism-spectrum disorders (*n* = 1), disorders of adult personality and behaviour (*n* = 1), or a combination of the above diagnoses (*n* = 12). No participant was acutely (physically or mentally) ill at the time of data collection. Except for 14 twin pairs, all DZ co-twins/co-siblings were of the same sex. The mean (SD) age difference between co-siblings was 3.6 (3.0) years.

A narrative description and rationale for the sample structure of the analysis is included in Appendix B (Supplementary Material).

### Neurocognitive Measures

Full-Scale IQ was measured using the Wechsler Adult Intelligence Scale, third edition (WAIS-III),^30^ or the Wechsler Abbreviated Scale of Intelligence (WASI).^31^ Our analysis further included a range of computerized cognitive measures. Such measures have been argued to optimise the statistical power of cognitive trials by reducing measurement error, between-site variability, and patient attrition through increased tolerability.^32^ The CANTAB^28^ measures used included *spatial working memory* [Spatial Working Memory task (SWM): Between Errors and Strategy]; *visual recognition* [Pattern Recognition Memory task (PRM): Percent Correct and Mean Correct Latency]; *sustained attention and visual processing* [Rapid Visual Processing task (RVP): signal detection measure A Prime, a measure of sensitivity to the target]; *mental flexibility/set shifting* [Intra-Extra Dimensional Set Shifting task (IED): Total Trials and Total Errors, both measures adjusted for number of stages completed]; and *spatial planning*/*problem-solving* [Stockings of Cambridge (SOC): Problems Solved in minimum moves (across difficulty levels), Mean Initial Thinking Time (highest difficulty level), a measure of pre-planning before the first move, and Mean Subsequent Thinking Time (highest difficulty level), a measure of execution planning after the first move]. Data on SOC and IED Total Errors were not available for the BD samples and were only analyzed in relation to SZ and the broad phenotype. Supplementary Table S2 summarises the contribution of each participating center to the IQ and CANTAB variables that were included in the model-fitting analysis. The means and SDs of the raw neurocognitive scores in the affected and unaffected subgroups are presented in Supplementary Table S3.

### Twin and Sibling Model-Fitting Analysis

The differential correlation of monozygotic (MZ) and dizygotic (DZ) twin (or sibling) pairs is utilized to estimate the effects of latent genetic and environmental factors on the variation of a trait. This is accomplished by fitting a model to the data in which the variation within and co-variation between twins/siblings is specified in terms of latent additive genetic effects (A), shared environmental effects (C), and non-shared (unique) environmental effects (E) including measurement error. The model is based on biometrical genetic theory, hypothesizing the correlation between MZ twins to be due to a 100% sharing of their genetic makeup and to everything else in the environment that makes them alike (C), and the correlation between DZ co-twins/siblings to be due to ~50% sharing of genes identical-by-descent and C.^33^ Non-twin siblings share the same expected correlations for A and C as DZ twins, although there might be additional twin-specific correlation due to factors like age. The differential MZ and DZ correlations across twins/siblings within traits allow for statistical power to estimate the effects of A, C (and E by deduction).

### Bivariate Twin and Sibling Modeling

The model above can be extended to a bivariate model. By using the differential MZ and DZ correlations across twins/siblings and across traits, the genetic (*r*_g_), shared environmental (*r*_c_), and unique environmental (*r*_e_) correlations between two traits (e.g., SZ and neurocognitive variable) can be estimated. These estimates reflect the extent to which *the same* genetic, common environmental, and unique environmental effects, respectively, influence a particular disorder phenotype and neurocognition. Moreover, one can combine the information from *r*_g_, *r*_c,_ and *r*_e_ with the heritability (*h*^2^), *c*^2^ and *e*^2^ of each trait to compute the genetic (*r*_ph-a_), common environmental (*r*_ph-c_), and unique environmental (*r*_ph-e_) contributions to the total phenotypic correlation (*r*_ph_) between two traits (Supplementary Figure S1).

Model fitting was performed by structural equation modeling (SEM) in the program OpenMx^34,35^ using full information maximum likelihood estimation, where continuous (cognitive) and binary (disease status) measures were analyzed jointly assuming a liability threshold model^36^ to reflect the risk for each of the three diagnostic phenotypes (each modeled separately): SZ, BD-I and the broad psychosis/BD-I phenotype including any primary psychotic disorder and BD-I. The model incorporated the effects of age, sex, years of education, and research center as covariates, thus correcting for group differences in demographic characteristics and for site differences in methods and procedures (e.g., order of neurocognitive test administration). Since most, if not all, cognitive functioning is mediated by global intelligence,^37^ IQ was not partially out to avoid a loss of meaningful variation.

### Ascertainment Correction and Sensitivity Analysis

The four study samples we combined in our mega-analysis^19,26,27,29^ included twin and sibling pairs specifically selected for schizophrenia spectrum or bipolar disorder diagnoses. For this reason, the model parameters for each neuropsychiatric phenotype (including the critical threshold and heritability of the underlying liability) were not directly estimated in the original studies,^19,26,27,29^ but were fixed at published population values. The same limitation and methodological imperative applied to the present mega-analysis, but with two essential modifications: First, to facilitate, for the first time, a novel comparison across SZ, BD-I, and a broad psychosis/BD-I phenotype within the same study, our main analysis fixed the model parameters at estimates derived from the Maudsley Twin Psychosis Series.^38–40^ This clinical case register series can be linked with population risk for the phenotypes of interest, but primarily offered the advantage of a consistent frame of ascertainment and methodology for deriving the model parameters for our three diagnostic phenotypes (thus offering optimal comparability across the phenotypes in our mega-analysis). However, the Maudsley Twin Psychosis Series^38–40^ also relies on a non-epidemiological sampling frame, which merited a second modification: Acknowledging the importance of basing critical threshold and heritability estimates (both population parameters) on as large and representative samples as possible, we further conducted a sensitivity analysis. The latter fixed the model parameters for SZ and BD-I at estimates derived from the most comprehensive synthesis conducted to date—a meta-analysis of virtually all twin studies of complex traits published until 2012, including (but not limited to) epidemiological, population-based, and population register studies.^41^

In line with the above rationale, we applied models where selection was accounted for by adjusting the bivariate genetic model.^42^ This involved fixing all model parameters of the selection variable to conservative values reported from the Maudsley Twin Psychosis Series^38–40^ (SZ: *h*^2^ = 0.84, *c*^2^ = 0.00, *e*^2^ = 0.16; BD-I: *h*^2^ = 0.85, *c*^2^ = 0.00, *e*^2^ = 0.15; broad psychosis/BD-I phenotype: *h*^2^ = 0.90, *c*^2^ = 0.00, *e*^2^ = 0.10), and the threshold on the liability to the population prevalence of 0.75% for SZ,^38^ 0.32% for BD-I^39^ and 1.7% for the broad psychosis/BD-I phenotype.^40^ The parameters for the cognitive variables and the correlation paths with each diagnostic phenotype were freely estimated. The significance of parameters (i.e., the fit of a sub-model) was computed by likelihood ratio tests. More information on these models can be found in the articles by Rijsdijk et al.^43^ and Hall et al.^42^

As elaborated above, the Maudsley Twin Psychosis Series^38–40^ is methodologically limited by the use of a non-epidemiological sampling frame. We therefore examined the sensitivity of our findings to modifying the model parameters in line with the *h*^2^, *c*^2,^ and *e*^2^ values reported for SZ and BD-I in a comprehensive meta-analysis by Polderman and colleagues^41^: SZ: *h*^2^ = 0.77, *c*^2^ = 0.01, *e*^2^ = 0.21; BD-I: *h*^2^ = 0.76, *c*^2^ = 0.00, *e*^2^ = 0.24. The threshold on the liability was fixed to the population prevalence of 1% for both disorders.

### Correlations

To estimate the MZ Twin and DZ Twin/Sibling correlations for each cognitive variable and between each cognitive measure and each diagnostic phenotype, we fitted a constrained correlational model to the MZ and DZ/Sibling data to get for each phenotype: 1 Within-Twin/Sibling Cross-Trait Correlation (e.g., IQ with liability to BD), which was equal across all participants regardless of zygosity and birth order; 1 MZ and 1 DZ/Sibling Cross-Twin/Sibling Cross-Trait Correlation; and 1 MZ and 1 DZ/Sibling Cross-Twin/Sibling Within-Cognitive-Measure Correlation. In line with the correction for selection described above, the MZ and DZ/Sibling Cross-Twin/Sibling correlations were fixed to 0.84 (*h*^2^ = 0.84 + *c*^2^ = 0) and 0.42 (*.5h*^*2*^ = 0.42 + *c*^*2*^ = 0), respectively, for schizophrenia; to 0.85 (*h*^2^ = 0.85 + *c*^2^ = 0) and 0.425 (*.5h*^*2*^ = 0.425 + *c*^*2*^ = 0), respectively, for I bipolar disorder; and to 0.90 (*h*^2^ = 0.90 + *c*^2^ = 0) and 0.45 (*.5h*^*2*^ = 0.45 + *c*^*2*^ = 0), respectively, for the broad phenotype. For the sensitivity analysis, the MZ and DZ/Sibling Cross-Twin/Sibling correlations were fixed to 0.78 (*h*^2^ = 0.77 + *c*^2^ = 0.01) and 0.395 (*.5h*^*2*^ = 0.385 + *c*^*2*^ = 0.01), respectively, for schizophrenia; and to 0.76 (*h*^2^ = 0.76 + *c*^2^ = 0) and 0.38 (*.5h*^*2*^ = 0.38 + *c*^*2*^ = 0), respectively, for bipolar I disorder.

### Defining Neurocognitive Endophenotypes

To consider a neurocognitive variable as an endophenotype, we combined all the following criteria: (1) A statistically significant within-twin/sibling cross-trait correlation with the diagnostic phenotype, which is the phenotypic correlation or overlap. In the genetic model: (2) A statistically significant heritability (*h*^2^) higher than 10%.^44–46^ (3) A statistically significant genetic correlation with the diagnostic phenotype (*r*_g_). (4) A statistically significant phenotypic correlation with the diagnostic phenotype due to additive genetic effects (*r*_ph-a_). The statistic *r*_ph-a_ is the product of the genetic correlation between the phenotypes and the square root of their heritabilities; it, therefore, in addition to the overlap in genes, also considers the impact of those genes (genetic factors) on the phenotypes.

### Bipolar I Disorder and Psychosis

Between over half to two-thirds of individuals with bipolar disorders have a lifetime history of psychosis,^47^ which is thought to co-segregate with more severe cognitive impairment compared to the non-psychotic form.^48^ Our BD-I subset did not provide sufficient statistical power to perform separate genetic model fitting analyses for participants with, versus without, lifetime psychosis. However, for completeness and illustrative purposes, we performed pairwise neurocognitive comparisons across BD-I patients with lifetime psychosis, BD-I patients without lifetime psychosis, SZ patients, and unaffected participants.

## Results

### Group Comparisons in Demographic and Neurocognitive Characteristics

The SZ, BD-I, broad phenotype, and unaffected groups differed in gender, age, and years of education (*P* values ranging from *P* < .01 to *P* < .001). Post-hoc comparisons indicated that the SZ and broad phenotype groups were younger and had a higher proportion of males, and fewer years of education compared to the BD-I and/or unaffected groups (Table 1). All neurocognitive variables showed statistically significant differences across the SZ, BD-I, broad phenotype and unaffected groups (Supplementary Table S3).

Compared to twins/siblings from unaffected pairs (*n* = 622), unaffected co-twins/siblings from discordant pairs (*n* = 171) were statistically significantly younger and had a higher proportion of males and non-psychotic, non-bipolar disorders (Supplementary Table S4). Furthermore, a consistent (but mostly statistically non-significant) pattern emerged in relation to the neurocognitive variables (Supplementary Table S4): The unaffected co-twins/siblings of the discordant pairs performed statistically non-significantly faster but less accurately compared to the unaffected pairs, except for IQ, where the results reached statistical significance (Supplementary Table S4).

As mentioned previously, demographic (age, gender, education) and research site effects were covaried for in our genetic model fitting analyses.

### Bivariate Modeling Analysis: Twin/sibling Correlations

The within-twin/sibling cross-trait correlations and the cross-twin/sibling within-trait- and cross-trait correlations for the neurocognitive measures are presented in Supplementary Tables S5–S7. All statistically significant within-twin/sibling cross-trait correlations (95% CIs not including zero) indicated that each diagnostic phenotype was associated with cognitive deficits (Supplementary Tables S5–S7). IQ, SWM Between Errors, and RVP A Prime showed statistically significant within-twin/sibling cross-trait correlations with both schizophrenia (SZ) (*r* values ranging from |0.13| to |0.24|) and bipolar I disorder (BD-I) (*r* values ranging from |0.14| to |0.17|) (Supplementary Tables S5–S6). PRM Percent Correct, IED Total Errors, SOC Problems Solved in minimum moves, and SOC Subsequent Thinking Time further showed statistically significant within-twin cross-trait correlations with SZ (*r* values ranging from |0.12| to |0.18|) (Supplementary Table S5), while SWM Strategy, PRM Mean Correct Latency and IED Total Trials further showed statistically significant within-twin/sibling cross-trait correlations with BD-I (*r* values ranging from |0.11| to |0.12|) (Supplementary Table S6). With the exception of SOC Mean Initial Thinking Time, all neurocognitive measures showed significant within-twin/sibling cross-trait correlations with the broad psychosis/BD-I phenotype (*r* values ranging from |0.13| to |0.25|) (Supplementary Table S7). For each diagnostic phenotype, neurocognitive measures with statistically non-significant within-twin/sibling cross-trait correlations were excluded from the further (genetic) analysis. The cross-twin/sibling within-trait- and cross-trait correlations showed higher values for MZ twins than DZ twins/siblings for most variables, indicating genetic (co)variance (Supplementary Tables S5-S7).

### Additive Genetic (*h*^2^), Shared Environmental (*c*^2^), and Unique Environmental (*e*^2^) Effects on Neurocognition

The additive genetic (*h*^2^), shared environmental (*c*^2^), and unique environmental (*e*^2^) estimates for the neurocognitive measures, as predicted by the bivariate ACE genetic models, are presented in Table 2. With the exception of SOC Problems Solved in minimum moves, variables that showed statistically significant correlations with SZ also showed statistically significant heritabilities (*h*^2^), which were moderate to high (0.27–0.75) (Table 2). Of the six variables that showed significant correlations with BD-I, IQ, SWM Between Errors and RVP A Prime also showed statistically significant heritabilities, which were moderate to high (0.29–0.67) (Table 2). With the exception of SWM Strategy, PRM Mean Correct Latency and SOC Problems Solved in Minimum Moves (all showing *h*^2^ < 10% in the context of the broad phenotype), all neurocognitive measures that showed significant correlations with the broad psychosis/BD-I phenotype also showed significant heritabilities higher than 10%, including two substantial heritabilities (IQ: *h*^2^ = 0.72; SOC Subsequent Thinking Time: *h*^2^ = 0.53) (Table 2). Shared environment (*c*^2^) did not explain inter-individual differences to a statistically significant extent (except for PRM Mean Correct Latency in all diagnostic phenotypes, and SOC Problems Solved in minimum moves in SZ), whereas individual-specific environmental effects (*e*^2^) accounted for a significant portion of the variance in all measures and phenotypes (Table 2).

**Table 2:** Additive Genetic (*h*^2^), Common Environmental (*c*^2^) and Unique Environmental (*e*^2^) Estimates for the Neurocognitive Measures Based on the Bivariate ACE Genetic Models with the Three Diagnostic Phenotypes ^a^

Cognitive measures ^b^	Schizophrenia			Bipolar I disorder			Broad psychosis/bipolar I disorder phenotype		
	h^2^ (95% CI)	c^2^ (95% CI)	e^2^ (95% CI)	h^2^ (95% CI)	c^2^ (95% CI)	e^2^ (95% CI)	h^2^ (95% CI)	c^2^ (95% CI)	e^2^ (95% CI)
IQ	**0.75** (0.52/ 0.8)	0.01 (0/ 0.24)	**0.24** (0.2/ 0.29)	**0.67** (0.45/ 0.8)	0.09 (0/ 0.3)	**0.24** (0.2/ 0.29)	**0.72** (0.49/ 0.79)	0.04 (0/ 0.25)	**0.25** (0.21/ 0.29)
Spatial working memory—between errors	**0.28** (0.02/ 0.59)	0.25 (0/ 0.49)	**0.47** (0.38/ 0.58)	**0.29** (0.01/ 0.59)	0.24 (0/ 0.49)	**0.47** (0.38/ 0.58)	**0.3** (0.03/ 0.59)	0.22 (0/ 0.46)	**0.48** (0.39/ 0.59)
Spatial working memory—strategy	Within-twin/sibling cross-trait correlation statistically non-significant ^c^ (Table S5[/scolor])			0.08 (0/ 0.44)	0.29 (0/ 0.43)	**0.63** (0.51/ 0.74)	**0.09** (0.01/ 0.44)	0.29 (0/ 0.42)	**0.63** (0.51/ 0.74)
Pattern recognition memory—mean correct latency	Within-twin/sibling cross-trait correlation statistically non-significant ^c^ (Table S5[/scolor])			0 (NA/ 0.02)	**0.46** (0.14/ 0.56)	**0.54** (0.44/ 0.64)	0.01 (0/ 0.04)	**0.45** (0.09/ 0.55)	**0.55** (0.44/ 0.65)
Pattern recognition memory—percent correct	**0.42** (0.02/ 0.66)	0.13 (NA/ 0.53)	**0.44** (0.34/ 0.57)	Within-twin/sibling cross-trait correlation statistically non-significant ^c^ (Table S6[/scolor])			**0.45** (0.02/ 0.65	0.1 (NA/ 0.52)	**0.45** (0.35/ 0.58)
Rapid visual processing—a prime	**0.38** (0.02/ 0.58)	0.1 (NA/ 0.41)	**0.53** (0.42/ 0.64)	**0.44** (0.06/ 0.58)	0.04 (0/ 0.37)	**0.52** (0.42/ 0.63)	**0.42** (0.05/ 0.57)	0.05 (0/ 0.35)	**0.53** (0.43/ 0.65)
Intra/extra-dimensional shift—total trials adjusted	Within-twin/sibling cross-trait correlation statistically non-significant ^c^ (Table S5[/scolor])			0.26 (0/ 0.38)	0 (0/ 0.26)	**0.74** (0.62/ 0.88)	**0.26** (0.01/ 0.38)	0 (0/ 0.26)	**0.74** (0.62/ 0.87)
Intra/extra-dimensional shift—total errors adjusted	**0.27** (0.01/ 0.45)	0 (0/ 0.22)	**0.73** (0.55/ 0.92)	Data not available			**0.27** (0.02/ 0.44)	0 (0/ 0.21)	**0.73** (0.56/ 0.92)
Stockings of Cambridge—minimum moves	0.02 (0/ 0.47)	**0.44** (0.04/ 0.56)	**0.53** (0.42/ 0.66)	Data not available			**0.04** (0.01/ 0.5)	0.41 (0/ 0.54)	**0.55** (0.43/ 0.68)
Stockings of Cambridge—initial thinking time	Within-twin/sibling cross-trait correlation statistically non-significant ^c^ (Table S5[/scolor])			Data not available			Within-twin/sibling cross-trait correlation statistically non-significant ^c^ (Table S7[/scolor])		
Stockings of Cambridge—subsequent thinking time	**0.52** (0.13/ 0.73)	0.11 (0/ 0.43)	**0.37** (0.27/ 0.52)	Data not available			**0.53** (0.14/ 0.72)	0.08 (0/ 0.41)	**0.38** (0.28/ 0.54)

^a^  *h*^2^, *c*^2^ and *e*^2^ indicate heritability, shared environmental and unique environmental effects, respectively. Results in bold have a statistically significant point estimate (the 95% confidence interval excludes 0). Only results for neurocognitive measures that showed statistically significant within-twin/sibling cross-trait correlations with the diagnostic phenotype are reported in the table.^b^ Higher cognitive scores indicate better performance in relation to IQ, Pattern Recognition Memory—Percent Correct, Rapid Visual Processing—A Prime and Stockings of Cambridge—Minimum Moves, and worse performance in relation to all remaining measures.

^c^ The measure was excluded from further (genetic) analysis.

### A, C, and E Overlap Between the Three Diagnostic Phenotypes and Neurocognition

The phenotypic correlations of the three diagnostic phenotypes with the neurocognitive measures and the decomposed sources of these correlations as predicted by the bivariate ACE models are presented in Tables 3–5. The A, C, and E correlations between the diagnostic phenotypes and the neurocognitive variables are presented in Tables 3–5 and in Supplementary Figures S2–S3. This information is presented only for measures with both statistically significant heritabilities exceeding 10% and statistically significant within-twin/sibling cross-trait correlations with one or more diagnostic phenotypes. Figure S4 presents the genetic correlations between the three diagnostic phenotypes and cognitive measures with heritabilities > 10%. Total phenotypic correlations (*r*_ph_) ranged from |0.13| to |0.23| for SZ, from |0.14| to |0.17| for BD-I, and from |0.17| to |0.25| for the broad diagnostic phenotype, suggesting that liability to each phenotype was associated with poorer performance in the respective neurocognitive measures (Tables 3–5). Five variables (IQ, SWM Between Errors, PRM Percentage Correct, IED Total Errors, SOC Mean Subsequent Thinking Time) showed low to moderate, statistically significant, genetic correlations (*r*_g_) with SZ (ranging from -0.18 to 0.36) (Table 3, Supplementary Figures S2, S4). Two variables (IQ, SWM Between Errors) showed moderate, statistically significant genetic correlations with BD-I (ranging from -0.20 to 0.24) (Table 4, Supplementary Figures S2–S4). Seven variables (IQ, SWM Between Errors, PRM Percentage Correct, RVP A Prime, IED Total Trials, IED Total Errors, SOC Mean Subsequent Thinking Time) showed statistically significant genetic correlations with the broad psychosis/BD-I phenotype, which ranged from moderate to high (−0.26 to 0.42) (Table 5, Supplementary Figure S4). All the above measures further showed statistically significant phenotypic correlations due to additive genetic effects (*r*_ph-a_) with the respective phenotypes (Tables 3–5). These ranged from |0.13| to |0.19| for SZ, from |0.12| to |0.15| for BD-I, and from |0.15| to |0.26| for the broad psychosis/BD-I phenotype. (Tables 3–5). These findings suggest that the liabilities to SZ, BD-I, and the broad psychosis/BD-I phenotype are significantly associated with poorer performance in all neurocognitive measures examined and that this association is significantly accounted for by additive genetic effects.

**Table 3:** —**Schizophrenia—**Phenotypic Correlations with Neurocognitive Measures, Decomposed Sources of Correlations as Predicted by the Bivariate ACE models, and A, C, and E Correlation Estimates ^a^

Cognitive measures ^b^	*r*_ph-a_ (95% CI)	*r*_ph-c_ (95% CI)	*r*_ph-e_ (95% CI)	*r*_ph_ (95% CI)	*r*_g_ (95% CI)	*r*_c_ (95% CI)	*r*_e_ (95% CI)
** IQ** ^†^	-**0.15** (-0.23/ -0.07)	0 (-0.02/ 0.01)	-**0.09** (-0.13/ -0.04)	-**0.23** (-0.3/ -0.16)	**0.18** (-0.28/ -0.09)	-1 (NA/ 1)	-**0.44** (-0.63/ -0.21)
** Spatial working memory –** **between errors** ^†^	**0.18** (0.08/ 0.28)	-0.02 (-0.02/ 0.02)	0.04 (-0.03/ 0.1)	**0.2** (0.11/ 0.28)	**0.36** (0.14/ 1)	-1 (NA/ 1)	0.14 (-0.11/ 0.37)
Spatial working memory – strategy	Within-twin/sibling cross-trait correlation statistically non-significant (Table S5[/scolor]) ^c^						
Pattern recognition memory—mean correct latency	Within-twin/sibling cross-trait correlation statistically non-significant (Table S5[/scolor]) ^c^						
** Pattern recognition memory—percent correct** ^†^	-**0.2** (-0.33/ -0.05)	-0.01 (-0.02/ 0.02)	0.03 (-0.11/ 0.16)	-**0.18** (-0.3/ -0.05)	-**0.33** (-1/ -0.09)	-1 (NA/ 1)	0.1 (-0.43/ 0.58)
Rapid visual processing – a prime	-**0.13** (-0.24/ -0.01)	0.01 (-0.02/ 0.02)	-0.01 (-0.1/ 0.08)	-**0.13** (-0.24/ -0.01)	-0.23 (-1/ 0.01)	1 (-1/ 1)	-0.04 (-0.34/ 0.26)
Intra/extra-dimensional shift – total trials adjusted	Within-twin/sibling cross-trait correlation statistically non-significant (Table S5[/scolor]) ^c^						
** Intra/extra-dimensional shift—total errors adjusted** ^†^	**0.15** (0.03/ 0.26)	0 (-0.01/ 0.01)	0 (-0.11/ 0.1)	**0.14** (0.04/ 0.24)	**0.3** (0.06/ 1)	-1 (-1/ 1)	-0.01 (-0.31/ 0.28)
Stockings of Cambridge – minimum moves	Heritability (*h*^2^) statistically non-significant and/or < 10% (Table 1) ^d^						
Stockings of Cambridge – initial thinking time	Within-twin/sibling cross-trait correlation statistically non-significant (Supplementary Table S5[/scolor]) ^c^						
** Stockings of Cambridge—subsequent thinking time** ^†^	**0.19** (0.06/ 0.31)	-0.01 (-0.02/ 0.02)	-0.04 (-0.12/ 0.04)	**0.13** (0.02/ 0.24)	**0.28** (0.28/ 0.64)	-1 (-1/ 1)	-0.18 (-0.48/ 0.14)

^a^ Point estimates (95% CI) are reported in the Table for measures that showed both a statistically significant within-twin/sibling cross-trait correlation with the diagnostic phenotype and a statistically significant heritability higher than 10%. The a and g subscripts in ***r***_**ph-a**_ and***r***_**g**_ both refer to additive genetic effects.

^b^ Higher cognitive scores indicate better performance in relation to IQ, Pattern Recognition Memory—Percent Correct, Rapid Visual Processing—A Prime and Stockings of Cambridge—Minimum Moves, and worse performance in relation to all remaining measures.

^c^ The measure was excluded from further (genetic) analysis.

^d^  ***r*ph-a** (95% CI) = -0.13 (-0.25/ -0.01);***r*ph-c** (95% CI) = -0.02 (-0.02/ -0.02);***r*ph-e** (95% CI) = -0.01 (-0.11/ 0.08); ***r***_**ph**_ (95% CI) = -0.17 (0.27/ 0.07);***r*g** (95% CI) = -1 (NA/-0.92); ***r*c (95% CI)** = -1 (NA/ 1); ***r*e (95% CI)** = -0.05 (-0.36/ 0.26).

^†^ The measure satisfied all criteria for endophenotypes.

**Table 4:** —**Bipolar I Disorder—**Phenotypic Correlations With Neurocognitive Measures, Decomposed Sources of Correlations as Predicted by the Bivariate ACE Models, and A, C, and E Correlation Estimates ^a^

Cognitive measures ^b^	*r*_ph-a_ (95% CI)	*r*_ph-c_ (95% CI)	*r*_ph-e_ (95% CI)	*r*_ph_ (95% CI)	*r*_g_ (95% CI)	*r*_c_ (95% CI)	*r*_e_ (95% CI)
** IQ** ^†^	-**0.15** (-0.24/ -0.07)	0.01 (-0.02/ 0.02)	0 (-0.05/ 0.05)	-**0.14** (-0.22/ -0.06)	-**0.2** (-0.34/ -0.08	1 (-1/ 1)	0.02 (-0.24/ 0.28)
** Spatial Working Memory –** **Between Errors** ^†^	**0.12** (0.03/ 0.21)	-0.02 (-0.02/ 0.02)	0.07 (-0.01/ 0.14)	**0.17** (0.09/ 0.25)	**0.24** (0.05/ 1)	-1 (NA/ 1)	0.25 (-0.02/ 0.51)
Spatial Working Memory – Strategy	Heritability (*h*^2^) statistically non-significant and/or < 10% (Table 1) ^c^						
Pattern Recognition Memory—Mean Correct Latency	Heritability (*h*^2^) statistically non-significant and/or < 10% (Table 1) ^d^						
Pattern Recognition Memory—Percent Correct	Within-twin/sibling cross-trait correlation statistically non-significant (Table S6[/scolor]) ^e^						
Rapid Visual Processing – A Prime	-0.08 (-0.19/ 0.03)	-0.01 (-0.02/ 0.02)	-0.08 (-0.16/ 0.01)	-**0.16** (-0.25/ -0.07)	-0.12 (-0.22/ 0.05)	-1 (NA/ 1)	-0.29 (-0.58/ 0.03)
Intra/Extra-Dimensional Shift – Total Trials Adjusted	Heritability (*h*^2^) statistically non-significant and/or < 10% (Table 1) ^f^						

^a^ Point estimates (95% CI) are reported for measures that showed both a statistically significant within-twin/sibling cross-trait correlation with the diagnostic phenotype and a statistically significant heritability higher than 10%. The a and g subscripts in ***r***_**ph-a**_ and***r***_**g**_ both refer to additive genetic effects. Data were not available for Intra/Extra-Dimensional Shift-Total Errors Adjusted; Stockings of Cambridge-Problems Solved in Minimum Moves (across difficulty levels); Stockings of Cambridge- Mean Initial Thinking Time (highest difficulty level); and Stockings of Cambridge- Mean Subsequent Thinking Time (highest difficulty level).

^b^ Higher cognitive scores indicate better performance in relation to IQ, PRM-PC, RVP-A and SOC-MM, and worse performance in relation to all the remaining measures.

^c^  ***r*ph-a** (95% CI) = 0.11 (0.01/ 0.2);***r*ph-c** (95% CI) = -0.02 (-0.02/ 0.02);***r*ph-e** (95% CI) = 0.02 (-0.07/ 0.11); ***r***_**ph**_ (95% CI) = 0.11 (0.03/ 0.19);***r*g** (95% CI) = 0.4 (0.4/ 1); ***r*c** (95% CI) = -1 (NA/ 1); ***r*e** (95% CI) = 0.07 (-0.22/ 0.35).

^d^  ***r*ph-a** (95% CI) = 0.04 (-0.06/ 0.14);***r*ph-c** (95% CI) = -0.02 (-0.02/ -0.02);***r*ph-e** (95% CI) = 0.11 (0.03/ 0.18); ***r***_**ph**_ (95% CI) = 0.12 (0.03/ 0.21);***r*g** (95% CI) = 1 (-1/ 1); ***r*c** (95% CI) = -1 (NA/ 1); ***r*e** (95% CI) = 0.38 (0.11/ 0.62).

^e^ The measure was excluded from further (genetic) analysis.

^f^  ***r*ph-a** (95% CI) = 0.12 (0.01/ 0.23);***r*ph-c** (95% CI) = 0 (-0.02/ 0.02);***r*ph-e** (95% CI) = 0 (-0.1/ 0.11); ***r***_**ph**_ (95% CI) = 0.12 (0.03/ 0.21);***r*g** (95% CI) = 0.25 (0.25/ 1); ***r*c** (95% CI) = -1 (-1/ 1); ***r*e** (95% CI) = 0.01 (-0.3/ 0.32).

^†^ The measure satisfied all criteria for endophenotypes.

**Table 5:** —**Broad Psychosis/Bipolar I Disorder Phenotype**—Phenotypic correlations with neurocognitive measures, decomposed sources of correlations as predicted by the bivariate ACE models, and A, C and E correlation estimates ^a^

Cognitive measures ^b^	*r*_ph-a_ (95% CI)	*r*_ph-c_ (95% CI)	*r*_ph-e_ (95% CI)	*r*_ph_ (95% CI)	*r*_g_ (95% CI)	*r*_c_ (95% CI)	*r*_e_ (95% CI)
** IQ** ^†^	-**0.21** (-0.28/ -0.13)	0.01 (-0.02/ 0.02)	-**0.03** (-0.06/ -0.01)	-**0.24** (-0.3/ -0.17)	-**0.26** (-0.37/ -0.17)	1 (-1/ 1)	-**0.21** (-0.37/ -0.04)
** Spatial Working Memory –** **Between Errors** ^†^	**0.22** (0.14/ 0.29)	-0.01 (-0.02/ 0.02)	0.05 (0/ 0.09)	**0.25** (0.17/ 0.31)	**0.42** (0.22/ 1)	-1 (NA/ 1)	**0.21** (0.02/ 0.39)
Spatial Working Memory – Strategy	Heritability (*h*^2^) statistically non-significant and/or < 10% (Table 1) ^c^						
Pattern Recognition Memory—Mean Correct Latency	Heritability (*h*^2^) statistically non-significant and/or < 10% (Table 1) ^d^						
** Pattern Recognition Memory—Percent Correct** ^†^	-**0.17** (-0.29/ -0.07)	-0.01 (-0.02/ 0.02)	0.01 (-0.05/ 0.07)	-**0.17** (-0.26/ -0.08)	-**0.27** (-1/ -0.11)	-1 (NA/ 1)	0.06 (-0.21/ 0.33)
** Rapid Visual Processing –** **A Prime** ^†^	-**0.18** (-0.26/ -0.08)	0.01 (-0.02/ 0.02)	-0.03 (-0.08/ 0.02)	-**0.2** (-0.27/ -0.12)	-**0.29** (-0.96/ -0.13)	1 (-1/ NA)	-0.12 (-0.32/ 0.08)
** Intra/Extra-Dimensional Shift –** **Total Trials Adjusted** ^†^	**0.15** (0.07/ 0.24)	0 (-0.02/ 0.02)	0.02 (-0.03/ 0.08)	**0.18** (0.1/ 0.25)	**0.32** (0.14/ 1)	-1 (NA/ 1)	0.09 (-0.12/ 0.29)
** Intra/Extra-Dimensional Shift—Total Errors Adjusted** ^†^	**0.2** (0.09/ 0.31)	0 (-0.01/ 0.01)	0.01 (-0.06/ 0.09)	**0.22** (0.12/ 0.31)	**0.41** (0.18/ 1)	1 (-1/ 1)	0.05 (-0.21/ 0.31)
Stockings of Cambridge – Minimum Moves	Heritability (*h*^2^) statistically non-significant and/or < 10% (Table 1) ^e^						
Stockings of Cambridge – Initial Thinking Time	Within-twin/sibling cross-trait correlation statistically non-significant (Table S7[/scolor]) ^f^						
** Stockings of Cambridge—Subsequent Thinking Time** ^†^	**0.26** (0.13/ 0.37)	-0.01 (-0.02/ 0.02)	-0.03 (-0.09/ 0.02)	**0.21** (0.1/ 0.32)	**0.37** (0.17/ 0.85)	-1 (-1/ 1)	-0.18 (-0.45/ 0.12)

^a^ Point estimates (95% CI) are reported in the Table for measures that showed both a statistically significant within-twin/sibling cross-trait correlation with the diagnostic phenotype and a statistically significant heritability higher than 10%. The a and g subscripts in ***r***_**ph-a**_ and***r***_**g**_ both refer to additive genetic effects. Data were not available for IED-TEA, SOC-MM, SOC-ITT, SOC-STT in the Bipolar I Disorder sample.

^b^ Higher cognitive scores indicate better performance in relation to IQ, PRM-PC, RVP-A and SOC-MM, and worse performance in relation to all the remaining measures.

^c^  ***r*ph-a** (95% CI) = 0.15 (0.07/ 0.23);***r*ph-c** (95% CI) = -0.02 (-0.02/ 0.02);***r*ph-e** (95% CI) = 0 (-0.05/ 0.05); ***r***_**ph**_ (95% CI) = 0.13 (0.06/ 0.21);***r*g** (95% CI) = 0.53 (0.53/ 1); ***r*c** (95% CI) = -1 (NA/ 1); ***r*e** (95% CI) = 0.01 (-0.19/ 0.21).

^d^  ***r*ph-a** (95% CI) = 0.09 (-0.01/ 0.19);***r*ph-c** (95% CI) = -0.02 (-0.02/ -0.02);***r*ph-e** (95% CI) = 0.08 (0.03/ 0.14); ***r***_**ph**_ (95% CI) = 0.15 (0.06/ 0.24);***r*g** (95% CI) = 1 (-1/ 1); ***r*c** (95% CI) = -1 (-1/ 1); ***r*e** (95% CI) = 0.36 (0.11/ 0.58).

^e^  ***r*ph-a** (95% CI) = -0.19 (-0.31/ -0.08);***r*ph-c** (95% CI) = -0.02 (-0.02/ -0.02);***r*ph-e** (95% CI) = -0.02 (-0.08/ 0.04); ***r***_**ph**_ (95% CI) = -0.23 (-0.33/ -0.13);***r*g** (95% CI) = -1 (NA/ -0.22); ***r*c** (95% CI) = -1 (NA/ 1); ***r*e** (95% CI) = -0.08 (-0.34/ 0.17).

^f^ The measure was excluded from further (genetic) analysis.

^†^ The measure satisfied all criteria for endophenotypes.

No shared environmental (*r*_c_) or unique environmental (*r*_e_) correlations with the diagnostic phenotypes were significant, except for the *r*_e_ correlations of IQ with SZ (-0.44) and with the broad psychosis/BD-I phenotype (−0.21) and the *r*_e_ correlation of SWM Between Errors with the broad phenotype (0.21) (Tables 3–5, Supplementary Figure S2).

### Neurocognitive Endophenotypes for Schizophrenia (SZ), Bipolar I Dsorder (BD-I), and the Broad Psychosis/BD-I Phenotype

Of the measures that met all endophenotypic criteria in relation to one or more diagnostic phenotypes, 2 measures emerged as transdiagnostic endophenotypes: IQ and SWM Between Errors (Tables 3–5). These satisfied all endophenotypic criteria in relation to SZ, BD-I and the broad psychosis/BD-I phenotype (Tables 3–5). Furthermore, an inspection and comparison of the 95% CIs around the point estimates of *r*_ph_, *h*^2^, *r*_g_, and *r*_ph-a_ for both validated endophenotypes suggested significant overlap in the 95% CIs across SZ, BD-I and the broad psychosis/BD-I phenotype, i.e., no statistically significant differences at the *P* < .05 level.

Three additional measures were validated as endophenotypes for SZ and the broad psychosis/BD-I phenotype (PRM Percent Correct, IED Total Errors, and SOC Mean Subsequent Thinking Time) (Tables 3, 5). Two further measures were validated as endophenotypes for the broad psychosis/BD-I phenotype (RVP A Prime and IED Total Trials) (Table 5).

### Sensitivity Analysis

The results of the sensitivity analysis (used to examine the sensitivity of our findings to modifying the model parameters in line with the *h*^2^, *c*^2^, and *e*^2^ values reported for SZ and BD-I in a comprehensive meta-analysis of twin studies of complex traits)^41^ are presented in Supplementary Tables S8–S12. The sensitivity analysis gave rise to a closely comparable pattern of findings, endorsing the same endophenotypes in relation to each diagnostic phenotype as in the main analysis.

### Bipolar I Disorder and Psychosis

Supplementary Table S13 presents the means and SDs of neuropsychological variables in the two BD-I sub-groups with/without lifetime psychosis, along with pairwise comparisons across the BD-I sub-groups, participants with schizophrenia, and those unaffected by psychotic/bipolar disorders. Consistent differences emerged between one or more patient groups and the unaffected group, and between schizophrenia and one or more of the remaining participant groups, but no statistically significant differences were found between the two BD-I subgroups (Supplementary Table S13).

## Discussion

Using twin and sibling data from three research centres in the UK, Denmark, and the Netherlands, our mega-analysis identified two transdiagnostic endophenotypes—IQ and Spatial Working Memory (SWM) - for SZ, BD-I, and a broad phenotype consisting of SZ, other primary psychoses, and BD-I. Visual recognition and mental flexibility/set shifting were endorsed as endophenotypes for SZ, but not for BD-I. Spatial problem solving was only evaluated in SZ and the broad phenotype and emerged as an endophenotype for both. The validated endophenotypes for SZ and BD-I and, additionally, sustained attention/rapid visual processing, further met endophenotypic criteria for the broad phenotype. The emergent endophenotypes showed moderate to high heritabilities (26–75%), low to moderate phenotypic correlations (|0.13|-|0.25|), and moderate genetic correlations (|0.18|-|0.42|) with the diagnostic phenotypes. Each of the latter shared 3% to 18% of their heritability with the respective endophenotypes. The highest genetic sharing was observed in relation to SWM (BD-I 6%; SZ 13%; broad phenotype 18%).

Our findings align with a growing body of evidence that points to transdiagnostic biological vulnerability in psychiatric disorders, whose pathophysiological mechanisms are only beginning to be clarified.^1,2,4,5,10,15,40,49^ The Cross-Disorder Group of the Psychiatric Genomics Consortium^10^ mapped this transdiagnostic underpinning onto 136 pleiotropic loci within various genes with a heightened expression in the brain throughout the lifespan. The measured pleiotropic effects operated on pairs of inter-related disorders, of which SZ and bipolar disorder (BD) showed the highest genetic correlation (*r*_g_ = 0.70 ± 0.02).^10^ Twenty-three (*n* = 23) of the loci were significantly associated with at least four psychiatric disorders.^10^

SZ and BD-I showed comparable genetic correlations (*r*_g_) with IQ (SZ -0.18; BD-I -0.20). These estimates coincide with, or closely approximate, the *r*_g_ (−0.20 to −0.26) between SZ and general cognitive ability,^50^ performance IQ,^26^ premorbid IQ^24^ and childhood IQ^25^ reported in large GWASs employing polygenic risk score (PRS) analyses,^50^ in population-based studies performing genetic model fitting of twin/sibling data^24,26^ and in population-based studies conducting bivariate genome-wide complex trait and PRS analyses.^25^ However, some non-population-based twin studies have reported higher genetic correlations between SZ and IQ (*r*_g_ −0.46 to −0.75).^19,20^ Such variability across studies is likely to arise from age-dependent genetic effects of SZ on cognitive traits (reported to be non-significant before, but high and significant *after* the peak age of onset, supporting late neurodevelopmental models),^51^ and from differences in sampling frames or exclusion criteria. For example, to allow a fair evaluation of performance on a cognitively demanding battery, we only included participants with IQ=>70 in the present analysis. This may have interfered with the representation of different genetic subtypes of SZ in our sample. Indeed, results from a large genome-wide association study and a replication cohort show that SZ diagnoses aggregate over at least two SZ subtypes, one resembling high intelligence and BD, and the other being a cognitive disorder independent of BD,^52^ a view originally suggested by Murray and colleagues.^53^

In line with the above findings, Miskowiak et al.^14^ using hierarchical cluster analysis, identified three transdiagnostic neurocognitive subgroups in an extended cohort of patients with SZ or BD: A “relatively intact” subgroup (39%; predominantly BD), one displaying selective deficits, particularly in working memory and processing speed (33%; relatively equally distributed across SZ and BD), and one with global impairments (28%; mainly SZ).^14^ The subgroups were not explained by clinical symptoms or medication, suggesting neurodevelopmental origins.^14^

Our finding regarding the transdiagnostic endophenotypic status of SWM extends earlier findings.^26,27,54,55^ Recently emerging molecular targets of likely relevance to SWM and psychiatric illness include the genes NISCH^56^ and EPHX2,^57^ the nitric oxide synthase 1 adaptor protein (NOS1AP)^58^ and rare copy number variants (CNVs).^59^

Meta-analytic findings suggest that patients with BD show qualitatively similar, but better, cognitive performance than those with SZ.^12^ Similarly, a comprehensive overview of neuropsychological deficits among unaffected relatives of SZ or BD patients concluded that the genetic risk for SZ is linked to a more substantial pattern of cognitive deficits than that for BD.^54^ Our findings fully align with this literature. Of the candidate endophenotypes that were evaluated in both SZ and BD-I, twice as many were validated as endophenotypes for SZ. Individuals with BD-I had markedly higher IQ (mean 107) than those with SZ (mean 94), which did not differ from that of controls (mean 109). This pattern may arise from a combination of concordant and discordant genetic effects on BD and intelligence, and from a partially shared etiology between BD and SZ. Several findings support this explanation. In a study by Smeland and colleagues,^55^ 81% of the SZ risk alleles that were shared with intelligence were associated with *lower* IQ, while 75% of the BD risk alleles that were shared with intelligence were associated with *higher* IQ.^55^ In line with this evidence, both excellent school performance and the poorest school grades at age 16 have been reported to predict future BD.^60^ Furthermore, Bansal and colleagues^52^ were able to show that the consistent but puzzling finding of a slightly positive genetic correlation between SZ and educational attainment can be attributed entirely to its genetic overlap with BD. “Purging” results of this overlap produced a negative correlation with educational attainment for SZ and a positive one for BD.^52^

It appears that several findings herein and in previous research point to a potential differentiation between SZ and BD in how genetic vulnerability to each disorder relates to IQ. It would therefore be incorrect to assume that genetic correlations with the same cognitive measures underlie the same genetic variation in SZ and BD-I. Furthermore, it is useful to acknowledge the caveat of the historical disagreement in what constitutes intelligence,^61^ and how twin findings on the genetic architecture of intelligence can help address the “missing heritability” in psychiatric genetics.^62^

In keeping with earlier findings,^22,26^ visual recognition, spatial problem-solving, and mental flexibility/set shifting emerged as endophenotypes for SZ. In addition, the analysis of the broad diagnostic phenotype gave rise to an enriched picture—consisting of a higher number of endophenotypes compared to either SZ or BD-I in isolation. This pattern is likely to have resulted from transdiagnostic components of variable salience within the discrete diagnostic phenotypes, whose “signal” was amplified when they were pooled together in a higher-powered analysis. Our results suggest that using broad diagnostic categories in genetic research may offer enhanced power and useful leads for further, finer-grained analysis.

Even though environmental factors play an established role in major psychopathology, our *r*_ph-c_ estimates were uniformly negligible and statistically non-significant across diagnostic phenotypes. This finding suggests that the co-clustering of lower cognitive functioning with higher genetic liability to the neuropsychiatric phenotypes is unlikely to have arisen from shared environmental factors. This interesting finding echoes earlier reports. For example, Toulopoulou and colleagues^20^ concluded that environmental effects account for little phenotypic correlation between cognition and schizophrenia. The probable reason for this is the very low environmental influence on cognition in this disorder.^63^

Our investigation is the first multi-center twin and sibling study to use advanced model fitting techniques to identify transdiagnostic and disorder-specific neurocognitive endophenotypes for psychotic and bipolar disorders. The use of a broad neurocognitive battery, a relatively homogeneous subtype of BD (BD-I), the statistical control for demographic confounders, and the sensitivity analysis, which allowed higher confidence in the resulting findings, are additional strengths of the study. Conversely, the relatively broad 95% confidence intervals of our estimates suggest that a larger sample size would have improved precision and reduced the risk of type II errors. In addition, the imperfect overlap of methodologies (e.g., sampling frames) across the participating centers did not allow a more penetrative analysis of potentially confounding influences on our estimates, for example, differences in ethnic make-up or environmental factors, which can be sub-optimally matched or corrected in meta- and mega-analyses. For compelling methodological reasons (for a detailed discussion see Dennis et al.^64^), our analysis did not covary for IQ. IQ-corrected results (available upon request) would have lost their statistical significance and intuitiveness, but this is likely to be a result of over-correction and other statistical artifacts.^64^

At least 65% of the BD-I probands had a history of psychosis. A GWAS study of BD cases unselected for psychosis found the highest load of schizophrenia risk alleles in the bipolar type of schizoaffective disorder, followed by BD-I, followed by BD-II.^65^ Such evidence suggests genetic similarities between SZ and BD-I which transcend psychosis. However, including exclusively BD-I participants with psychotic features might have increased transdiagnostic similarities in our study. Our pairwise neuropsychological comparisons did not suggest that psychotic BD-I is most like schizophrenia. However, it is important to acknowledge the limitations of the non-epidemiological sampling frame in our study.

A final important note of caution draws on the ecologic validity and applicability of endophenotypic research to the real world, highlighting the implications of the accessibility of research laboratory tools for the translatability of theoretical advances into psychiatric clinical practice. Meaningful biotyping is likely to rely on combined information from different biological and biobehavioural terrains, as probed by various combinations of neuroimaging, neurocognitive, and genetic testing. These specialized tools are not routinely available to most patients. Importantly, while there are reciprocal links across endophenotypes, biotyping, classification, and treatment, biotypes depend on the exact measures being used and analyzed, and hence can be variable.

In conclusion, IQ and SWM are transdiagnostic endophenotypes for schizophrenia, bipolar I disorder, and mixed primary psychoses. The high likelihood that the two neurocognitive traits lie on the same genetic pathway to severe mental illness^26,66^ makes IQ and SWM promising candidates for the exploration of shared molecular mechanisms and of the joint action of sets of candidate genes in severe mental illness. Spatial problem-solving, visual recognition, and mental flexibility are endophenotypes for schizophrenia (while sustained attention/rapid visual processing was additionally validated as endophenotype for the broad phenotype) and merit further exploration as potential transdiagnostic traits. Using multiple endophenotypes that cut across psychotic and bipolar disorder diagnoses can help identify meaningful biotypes, aid genetic discovery and develop novel treatments. Our findings add to a growing body of evidence^1,2,4,5,10,15,49,52^ which spurs cautious optimism that a psychiatric nosology based on aetiology rather than pure phenotypical classifications may be feasible in the future.

## Supplementary material

Supplementary material is available at https://academic.oup.com/schizophreniabulletin.

sbaf050_suppl_Supplementary_Tables_S1-S13_Figures_S1-S4
