# Supplementary material for: Transdiagnostic Neurocognitive Endophenotypes for Schizophrenia, Bipolar I Disorder and a Broad Psychosis/Bipolar I Disorder Phenotype: A Mega-Analysis of Twin and Sibling Data
Source: Schizophr Bull. 2025 May 9;52(1):sbaf050. doi: 10.1093/schbul/sbaf050 (PMC12809861; doi:10.1093/schbul/sbaf050)
Supplement: sbaf050_suppl_Supplementary_Tables_S1-S13_Figures_S1-S4 [file sbaf050_suppl_supplementary_tables_s1-s13_figures_s1-s4.docx]

**Supplementary Tables**

**Table S1** Summary of methods in participating centres

| **Centre** | **United Kingdom - Schizophrenia** | **United Kingdom - Bipolar I Disorder** | **Denmark - Schizophrenia** | **Netherlands - Bipolar I Disorder** |
| --- | --- | --- | --- | --- |
| **Reference** | Toulopoulou et al., 2007 ^a^ | Georgiades et al., 2016 ^b^ | Lemvigh et al., 2022 ^c^ | Bootsman et al., 2015 ^d^ |
| **Country** | United Kingdom (UK) | United Kingdom (UK) | Denmark | Netherlands |
| **Index diagnosis** | Schizophrenia or schizoaffective disorder (DSM-IV) | Bipolar disorder or schizoaffective disorder–bipolar type (DSM-IV) | Schizophrenia-spectrum disorder (ICD-8, ICD-10) | Bipolar disorder (DSM-IV) |
| **Inclusion criteria** | Twins affected with or unaffected by schizophrenia or schizoaffective disorder (DSM-IV) | Twin pairs affected with or unaffected by bipolar or schizoaffective disorder (bipolar type) (DSM-IV) | Twin pairs concordant or discordant for a diagnosis in the schizophrenia spectrum (main or secondary lifetime diagnosis in ICD-8 of  295, 297, 298.29, 298.39, 298.89, 298.99, 299.05, 299.09, 301.09,  301.29, or in ICD-10 of F2x.x), and healthy control (HC) pairs. | Twin pairs affected with or unaffected by bipolar disorder (DSM-IV) |
| **Exclusion criteria** | Age younger than 18 years, a history of a neurological disorder or of a systemic illness with known neurological complications, a history of significant head injury associated with loss of consciousness for more than 1 minute, and current harmful substance use or dependence (defined as within the last 12 months). The control twins were additionally free of personal or family history of psychosis or schizophrenia spectrum disorder. | A first language other than English, age younger than 16 years/older than 67 years, IQ <70, a history of any disorder with known neurological symptoms or complications, and a history of head injury resulting in loss of consciousness for more than 10 min. The control participants were additionally free of personal and family histories, up to second-degree relatives, of bipolar and psychotic spectrum disorders. | Serious head trauma (recorded in the medical journal), drugs/alcohol addiction, serious physical illness, and pregnancy (due to MRI scans). HC pairs were excluded based on the presence of a diagnosis of major psychosis in any first-degree relatives (F2x.x, F30, F31, and F32.3). | History of drug or alcohol dependency for the last 6 months prior to inclusion in the study, and severe medical illness, verified with a medical history inventory. Control pairs had no history of Axis I or II disorders according to DSM-IV criteria (confirmed with the SCID and SIDP, respectively) and no history of severe medical illness. Furthermore, they had no first-degree relative with a history of a major Axis I psychiatric disorder (DSM-IV). |
| **Clinical Assessment** | DSM-IV diagnoses were made using the Schedule for Affective Disorders and Schizophrenia–Lifetime Version, supplemented by information from medical notes or by using the Structured Clinical Interview for DSM-IV. Psychotic symptoms in the probands in the month before testing were assessed using the Scale for the Assessment of Positive Symptoms (SAPS) and the Scale for the Assessment of Negative Symptoms (SANS). Medication status was recorded at the time of the assessment. | Diagnoses were based on structured clinical interviews using the Schedules for Clinical Assessment in Neuropsychiatry (SCAN 2.1), the Structured Clinical Interview for DSM-IV Axis I Disorders (SCID-I) or the Schedule for Affective Disorders and Schizophrenia – Lifetime Version (SADS-L). These were supplemented by information from medical notes (patients only). The Beck Depression Inventory (BDI)21 and the Altman Self-Rating Mania Scale (ASRM)22 were administered on the day of testing to assess current mood (all participants). Medication status was recorded at the time of the assessment. | Register diagnoses were verified according to ICD-10 criteria using the Schedules for Clinical Assessment in Neuropsychiatry (SCAN) interview. In cases of discrepancy between the register and project diagnosis, the project diagnosis was used. Psychopathology was rated using the Positive and Negative Symptom Scale (PANSS), Hamilton’s Depression (HAM-D17) and Anxiety (HAM-A14) scales, and the Bech-Rafaelsen Mania Scale (MAS). | Clinical diagnosis of Axis I psychiatric disorders and Axis II personality disorders was confirmed using the Structured Clinical Interview for DSM-IV (SCID) and the Structured Interview for DSM-IV Personality (SIDP), respectively, and through available medical records. Patients were also interviewed on their medication history. The current mood state of BD patients was assessed using the Young Mania Rating Scale (YMRS) and the Inventory for Depressive Symptomatology (IDS). |
| **Assessment of zygosity** | Zygosity was determined by assessment of 12 highly polymorphic microsatellite markers and a standardized twin likeness questionnaire. | Zygosity was preliminarily ascertained on the basis of a twin questionnaire. The results of the questionnaire were confirmed by DNA analysis of blood or cheek swab samples. DNA analysis was based on a set of 18 highly polymorphic markers (consisting of between 5 and 15 alleles and a mix of di-, tri- and tetranucleotide microsatellites). The results from each twin pair were compared to look for matching genotypes/alleles and a statistic calculated to determine the probability of the pair being MZ or DZ. | Zygosity was verified by blood samples. In cases where DNA was not available register-based information was used. | Zygosity was determined with DNA fingerprinting using high polymorphic microsatellite markers 9 to 11. |
| **Ethical approval and participant consent** | The study was approved by the UK Multicenter Research Ethics Committee and all of the subjects gave written informed consent before participating. | The study was approved by the ethical committee of the IoPPN, King’s College London. After complete description of the study to the participants, written informed consent was obtained. | The study was approved by The Danish Health and Medicines Authority, The Danish Data Protection Agency (2010-41-5468), and The Danish National Committee on Health Research Ethics (H-2-2010-128). Informed consent was obtained from all participants. | The study was approved by the medical ethics committee for research in humans of the University Medical Center Utrecht and was performed according to the directives of the Declaration of Helsinki (amendment of Edinburgh, 2000). All the twins participated after providing written informed consent. |

^a^ Additional references to the twin schizophrenia series in the UK: Owens et al, 2011a; Owens et al, 2011b; Owens et al, 2012

^b^ Additional references to the twin and sibling bipolar disorder series in the UK: McDonald et al, 2004; Schulze et al, 2011

^c^ Additional references to the twin schizophrenia series in Denmark: Legind et al, 2019; Lemvigh et al, 2020

^e^ Additional references to the twin bipolar disorder series in the Netherlands: van der Schot et al, 2009; van der Schot et al, 2010

References

Bootsman F, Brouwer RM, Schnack HG, et al. Genetic and environmental influences on cortical surface area and cortical thickness in bipolar disorder. *Psychol Med.* . 2015;45(1):193-204. doi:10.1017/S0033291714001251

Georgiades A, Rijsdijk F, Kane F, et al. New insights into the endophenotypic status of cognition in bipolar disorder: genetic modelling study of twins and siblings. *Br J Psychiatry*. . 2016;208(6):539-47. doi:10.1192/bjp.bp.115.167239

Legind CS, Broberg BV, Mandl RCW, et al. Heritability of cerebral glutamate levels and their association with schizophrenia spectrum disorders: a 1[H]-spectroscopy twin study. *Neuropsychopharmacology*. . 2019;44(3):581-589. doi:10.1038/s41386-018-0236-0

Lemvigh CK, Brouwer RM, Sahakian BJ, et al. Heritability of Memory Functions and Related Brain Volumes: A Schizophrenia Spectrum Study of 214 Twins. *Schizophrenia Bulletin Open*. 2020 Jan;1(1):sgaa066doi. doi: 10.1093/schizbullopen/sgaa066

Lemvigh CK, Brouwer RM, Pantelis C, et al. Heritability of specific cognitive functions and associations with schizophrenia spectrum disorders using CANTAB: a nation-wide twin study. *Psychol Med.* 2022 Apr;52(6):1101-1114. doi: 10.1017/S0033291720002858. Epub 2020 Aug 11. PMID: 32779562.

McDonald C, Bullmore ET, Sham PC, Chitnis X, Wickham H, Bramon E, et al. Association of genetic risks for schizophrenia and bipolar disorder with specific and generic brain structural endophenotypes. *Arch Gen Psychiatry* 2004; 61: 974–84.

Owens SF, Picchioni MM, Rijsdijk FV, et al. Genetic overlap between episodic memory deficits and schizophrenia: results from the Maudsley Twin Study. *Psychol Med*. . 2011a;41(3):521-32. doi:10.1017/S0033291710000942

Owens SF, Rijsdijk F, Picchioni MM, et al. Genetic overlap between schizophrenia and selective components of executive function. *Schizophr Res.* . 2011b;127(1-3):181-7. doi:10.1016/j.schres.2010.10.010

Owens SF, Picchioni MM, Ettinger U, et al. Prefrontal deviations in function but not volume are putative endophenotypes for schizophrenia*. Brain*. 2012 Jul;135(Pt 7):2231-44. doi: 10.1093/brain/aws138. Epub 2012 Jun 12. PMID: 22693145; PMCID: PMC3381723.

Schulze KK, Walshe M, Stahl D, Hall MH, Kravariti E, Morris R, et al. Executive functioning in familial bipolar I disorder patients and their unaffected relatives. *Bipolar Disord* 2011; 13: 208–16.

Toulopoulou T, Picchioni M, Rijsdijk F, et al. Substantial genetic overlap between neurocognition and schizophrenia: genetic modeling in twin samples. *Arch Gen Psychiatry*. . 2007;64(12):1348-55. doi:10.1001/archpsyc.64.12.1348

van der Schot AC, Vonk R, Brans RG, et al. Influence of genes and environment on brain volumes in twin pairs concordant and discordant for bipolar disorder*. Arch Gen Psychiatry*. . 2009;66(2):142-51. doi:10.1001/archgenpsychiatry.2008.541

van der Schot AC, Vonk R, Brouwer RM, et al. Genetic and environmental influences on focal brain density in bipolar disorder. *Brain*. . 2010;133(10):3080-92. doi:10.1093/brain/awq236

**Table S2** Number (%) of participants contributing analytic data from each centre

|  | **United Kingdom - SZ** | | **United Kingdom - BD-I** | | | **Denmark - SZ** | | | **Netherlands - BD-I** | | | **All centres** | | | |
| --- | --- | --- | --- | --- | --- | --- | --- | --- | --- | --- | --- | --- | --- | --- | --- |
|  | **Affected** | **Unaffected** | | **Affected** | **Unaffected** | | **Affected** | **Unaffected** | | **Affected** | **Unaffected** | | **Affected** | **Unaffected** | **Total** |
| **Cognitive measures** | **N (%)** | | **N (%)** | | | **N (%)** | | | **N (%)** | | | **N (%)** | | | **N (%)** |
|  |  |  | |  |  | |  |  | |  |  | |  |  |  |
| IQ | 115 (11) | 344 (33) | | 79 (7) | 247 (24) | | 49 (5) | 146 (14) | | 14 (1) | 55 (5) | | 257 (24) | 793 (76) | 1050 (100) |
| Spatial Working Memory – Between Errors | 37 (5) | 94 (13) | | 76 (11) | 245 (34) | | 49 (7) | 146 (20) | | 14 (2) | 56 (8) | | 176 (25) | 541 (75) | 717 (100) |
| Spatial Working Memory –  Strategy |  |  |  |  |  |  |  |  |  |  |  |  |  |  |  |
| Pattern Recognition Memory – Mean Correct Latency | 31 (8) | 55 (14) | | 75 (18) | 245 (60) | | - | - | | - | - | | 106 (26) | 300 (74) | 406 (100) |
| Pattern Recognition Memory – Percent Correct |  |  |  |  |  |  |  |  |  |  |  |  |  |  |  |
| Rapid Visual Processing –  A Prime | 36 (6) | 94 (15) | | 73 (11) | 245 (38) | | 46 (7) | 146 (23) | | - | - | | 155 (24) | 485 (76) | 640 (100) |
| Intra/Extra-Dimensional Shift – Total Trials Adjusted | 36 (5) | 94 (15) | | 76 (12) | 245 (38) | | 48 (7) | 146 (23) | | - | - | | 160 (25) | 485 (75) | 645 (100) |
| Intra/Extra- Dimensional Shift – Total Errors Adjusted | 36 (11) | 94 (29) | | - | - | | 48 (15) | 146 (45) | |  |  |  | 84 (26) | 240 (74) | 324 (100) |
| Stockings of Cambridge –  Minimum Moves | 37 (11) | 108 (32) | | - | - | | 47 (14) | 146 (43) | | - | - | | 84 (25) | 254 (75) | 338 (100) |
| Stockings of Cambridge –  Initial Thinking Time |  |  |  |  |  |  |  |  |  |  |  |  |  |  |  |
| Stockings of Cambridge – Subsequent Thinking Time |  |  |  |  |  |  |  |  |  |  |  |  |  |  |  |

**Table S3** Mean (SD) neurocognitive scores in affected and unaffected participants

|  | **Schizophrenia (SZ) (n=145)** | **Bipolar I Disorder (BD-I) (n=86)** | **Broad Psychosis/BD-I phenotype (n=257)** | **Unaffected (n=793)** |
| --- | --- | --- | --- | --- |
|  | **(Group 1)** | **(Group 2)** | **(Group 3)** | **(Group 4)** |
| **Cognitive measures ^a^** | **Mean (SD)** | **Mean (SD)** | **Mean (SD)** | **Mean (SD)** |
|  |  |  |  |  |
| IQ ^b^ | 94.06 (14.23) | 107.44 (13.01) | 100.19 (14.91) | 108.95 (13.74) |
| Spatial Working Memory –  Between Errors ^c^ | 36.34 (26.8) | 32.93 (20.64) | 32.73 (23.14) | 21.26 (17.36) |
| Spatial Working Memory –  Strategy ^d^ | 32.57 (7.46) | 33.68 (5.56) | 32.86 (6.56) | 30.92 (6.40) |
| Pattern Recognition Memory –  Mean Correct Latency ^e^ | 2572.63 (1016.43) | 2267.99 (625.99) | 2358.98 (764.46) | 2056 (523.94) |
| Pattern Recognition Memory –  Percent Correct ^f^ | 74.87 (19.68) | 88.50 (10.41) | 84.63 (14.97) | 90.99 (8.47) |
| Rapid Visual Processing –  A Prime ^g^ | 0.87 (0.07) | 0.89 (0.05) | 0.88 (0.06) | 0.91 (0.05) |
| Intra/Extra-Dimensional Shift –  Total Trials Adjusted ^h^ | 143.81 (95.96) | 95.64 (30.48) | 117.02 (68.25) | 92.65 (42.22) |
| Intra/Extra- Dimensional Shift –  Total Errors Adjusted ^i^ | 51.29 (53.9) | - | 47.83 (47.87) | 24.5 (23.49) |
| Stockings of Cambridge –  Minimum Moves ^j^ | 7.77 (2.5) | - | 8.05 (2.38) | 9.41 (1.75) |
| Stockings of Cambridge –  Initial Thinking Time ^k^ | 11509.78 (8451.86) | - | 11689.83 (7976.88) | 13786.18 (8797.63) |
| Stockings of Cambridge –  Subsequent Thinking Time ^l^ | 3115.71 (4993.15) | - | 2556.53 (4412.99) | 1061.37 (2015) |

^a^ Higher cognitive scores indicate better performance in relation to IQ, Pattern Recognition Memory – Percent Correct, Rapid Visual Processing - Prime A, Stockings of Cambridge – Minimum Moves, and worse performance in relation to all the remaining measures. Statistical group comparisons in neurocognitive variables were performed using linear regression analysis with robust standard errors and with the cluster option to account for the dependency of observations within twin or sibling pairs (clusters). The degrees of freedom in these models refer to the number of clusters rather than participants. Statistical results with P values < 0.004 survive correction for multiple comparisons (Statistical threshold of 0.05 divided by 11 cognitive variables = 0.0045).

^b^ Group 1, Group 2, Group 4: F(2, 490)=42.31, P<0.001: Group 1 < Group 2, Group 4 | Group 3, Group 4: F(1, 493)=50.09, P<0.001: Group 3 < Group 4

^c^ Group 1, Group 2, Group 4: F(2, 385)=20.52, P<0.001: Group 1, Group 2 > Group 4 | Group 3, Group 4: F(1, 388)=35.49, P<0.001: Group 3 > Group 4

^d^ Group 1, Group 2, Group 4: F(2, 385)=9.56, P<0.001: Group 2 > Group 4 | Group 3, Group 4: F(1, 388)=12.19, P<0.001: Group 3 > Group 4

^e^ Group 1, Group 2, Group 4: F(2, 212)=5.91, P<0.01: Group 1, Group 2 > Group 4 | Group 3, Group 4: F(1, 212)=11.31, P<0.001: Group 3 > Group 4

^f^ Group 1, Group 2, Group 4: F(2, 212)=8.57, P<0.001: Group 1 < Group 2, Group 4 | Group 3, Group 4: F(1, 212)=12.22, P<0.001: Group 3 < Group 4

^g^ Group 1, Group 2, Group 4: F(2, 335)=13.97, P<0.001: Group 1, Group 2 < Group 4 | Group 3, Group 4: F(1, 339)=29.84, P<0.001: Group 3 < Group 4

^h^ Group 1, Group 2, Group 4: F(2, 336)=7.80, P<0.001: Group 1 > Group 2, Group 4 | Group 3, Group 4: F(1, 339)=16.24, P<0.001: Group 3 > Group 4

^i^ Group 1, Group 4: F(1, 171)=13.13, P<0.001: Group 1 > Group 4 | Group 3, Group 4: F(1, 174)=17.28, P<0.001: Group 3 > Group 4

^j^ Group 1, Group 4: F(1, 178)=18.90, P<0.001: Group 1 < Group 4 | Group 3, Group 4: F(1, 181)=18.89, P<0.001: Group 3 < Group 4

^k^ Group 1, Group 4: F(1, 178)=3.45, ns | Group 3, Group 4: F(1, 181)=3.94, P<0.05: Group 3 < Group 4

^l^ Group 1, Group 4: F(1, 178)=8.37, P<0.01: Group 1 > Group 4; | Group 3, Group 4: F(1, 181)=7.30, P<0.01: Group 3 > Group 4

**Table S4** Comparison of unaffected twins/siblings from discordant pairs versus unaffected pairs ^a^

|  |  | | |  |
| --- | --- | --- | --- | --- |
|  | **Unaffected Twins and Siblings (n=793)** | | |  |
|  |  |  |  |  |
|  | **From Discordant Pairs**  **(n=171)** |  | **From Unaffected Pairs**  **(n=622)** | **Statistical comparison ^a^** |
|  |  |  |  |  |
| **Demographic characteristics** |  |  |  |  |
| Gender, n (%) |  |  |  | Wald chi2(1)=15.87, *P*<0.001 |
| Men | 62 (48.1%) |  | 175 (28.1%) |  |
| Women | 67 (51.9%) |  | 447 (71.9%) |  |
| Age, years: Mean (s.d.) | 38.59 (11.34) |  | 42.60 (12.12) | F(1, 439)=11.00, *P*<0.001 |
| Education, years: mean (s.d.) | 14.65 (2.92) |  | 14.53 (2.80) | ns |
| Non-psychotic, non-bipolar psychiatric disorders, n (%) | 34 (5.5%) |  | 41 (31.8%) | Wald chi2(1)=56.65, *P*<0.001 |
|  |  |  |  |  |
| **Neurocognitive characteristics ^b^** |  |  |  |  |
| IQ | 106.84 (13.29) |  | 110.17 (13.28) | F(1, 385)=5.09, *P*<0.05 |
| Spatial Working Memory – Between Errors | 22.38 (17.80) |  | 20.78 (17.33) | ns |
| Spatial Working Memory – Strategy | 30.84 (6.62) |  | 30.75 (6.39) | ns |
| Pattern Recognition Memory – Mean Correct Latency | 1994.48 (582.40) |  | 2064.42 (505.16) | ns |
| Pattern Recognition Memory – Percent Correct | 89.02 (10.07) |  | 91.51 (8.02) | ns |
| Rapid Visual Processing – A Prime | 0.91 (0.05) |  | 0.92 (0.047) | ns |
| Intra/Extra-Dimensional Shift – Total Trials Adjusted | 97.30 (50.57) |  | 91.34 (39.94) | ns |
| Intra/Extra- Dimensional Shift – Total Errors Adjusted | 30.47 (34.02) |  | 22.29 (18.50) | ns |
| Stockings of Cambridge – Minimum Moves | 9.16 (1.56) |  | 9.54 (1.82) | ns |
| Stockings of Cambridge – Initial Thinking Time | 13531.46 (8245.51) |  | 13961.31 (8960.44) | ns |
| Stockings of Cambridge – Subsequent Thinking Time | 1007.26 (1342.59) |  | 1109.09 (2237.59) | ns |
|  |  |  |  |  |

^a^ The two groups were compared using logistic or linear regression analysis with robust standard errors and with the cluster option to account for the dependency of observations within twin or sibling pairs (clusters). The degrees of freedom in these models refer to the number of clusters rather than participants.

^b^ Higher cognitive scores indicate better performance in relation to IQ, Pattern Recognition Memory – Percent Correct, Rapid Visual Processing - Prime A, Stockings of Cambridge – Minimum Moves, and worse performance in relation to all the remaining measures. Of the neurocognitive comparisons, the only statistically significant result [for IQ: F(1, 385)=5.09, P<0.05] does not survive correction for multiple comparisons (Statistical threshold of 0.05 divided by 11 cognitive variables = 0.0045).

**Table S5 - Schizophrenia** - Within-twin/sibling cross-trait correlations, cross-twin/sibling within-trait correlations and cross-twin/sibling cross-trait correlations based on a constrained correlation (non-genetic) model

| **Cognitive measures ^a^** | ***Within-Twin/Sibling Cross-Trait Correlations*** | ***Cross-Twin/Sibling Within-Trait Correlations*** | | ***Cross-Twin/Sibling Cross-Trait Correlations*** | |
| --- | --- | --- | --- | --- | --- |
|  |  | ***Monozygotic (MZ)*** | ***Dizygotic (DZ)*** | ***Monozygotic (MZ)*** | ***Dizygotic (DZ)*** |
| IQ | -**0.24**  (-0.3/ -0.17) | **0.76**  (0.71/ 0.8) | **0.39**  (0.25/ 0.5) | -**0.15**  (-0.22/ -0.07) | -0.1  (-0.21/ 0.01) |
| Spatial Working Memory –  Between Errors | **0.19**  (0.1/ 0.28) | **0.53**  (0.42/ 0.62) | **0.38**  (0.23/ 0.5) | **0.16**  (0.06/ 0.26) | 0  (-0.14/ 0.13) |
| Spatial Working Memory –  Strategy | 0.05  (-0.05/ 0.14) | **0.39**  (0.26/ 0.5) | **0.33**  (0.19/ 0.46) | **0.15**  (0.03/ 0.25) | -0.06  (-0.19/ 0.08) |
| Pattern Recognition Memory –  Mean Correct Latency | 0.12  (-0.01/ 0.25) | **0.44**  (0.3/ 0.56) | **0.45**  (0.21/ 0.61) | 0.06  (-0.08/ 0.2) | 0.01  (-0.24/ 0.25) |
| Pattern Recognition Memory –  Percent Correct | -**0.18**  (-0.31/ -0.05) | **0.56**  (0.43/ 0.66) | **0.35**  (0.05/ 0.55) | -**0.2**  (-0.34/ -0.06) | -0.14  (-0.39/ 0.13) |
| Rapid Visual Processing –  A Prime | -**0.13**  (-0.22/ -0.03) | **0.47**  (0.35/ 0.58) | **0.25**  (0.06/ 0.41) | -**0.14**  (-0.24/ -0.02) | 0.04  (-0.11/ 0.19) |
| Intra/Extra-Dimensional Shift –  Total Trials Adjusted | 0.09  (0/0.18) | **0.28**  (0.14/ 0.41) | 0.09  (-0.09/ 0.27) | 0.08  (-0.02/ 0.19) | -0.06  (-0.2/ 0.09 ) |
| Intra/Extra- Dimensional Shift–  Total Errors Adjusted | **0.14**  (0.04/ 0.24) | **0.34**  (0.15/ 0.5) | -0.18  (-0.43/ 0.11) | **0.15**  (0.03/ 0.27) | 0.02  (-0.13/ 0.17) |
| Stockings of Cambridge –  Minimum Moves | -**0.18**  (-0.28/ -0.07) | **0.46**  (0.28/ 0.6) | **0.48**  (0.27/ 0.63) | -**0.15**  (-0.28/ -0.03) | -0.12  (-0.26/ 0.03) |
| Stockings of Cambridge –  Initial Thinking Time | -0.06  (-0.16/ 0.04) | -0.13  (-0.34/ 0.08) | -0.1  (-0.34/ 0.16) | -0.01  (-0.12/ 0.11) | 0.09  (-0.08/ 0.25) |
| Stockings of Cambridge –  Subsequent Thinking Time | **0.12**  (0.01/ 0.23) | **0.62**  (0.47/ 0.73) | **0.35**  (0.16/ 0.51) | **0.18**  (0.06/ 0.3) | 0  (-0.15/ 0.14) |

^a^ Higher cognitive scores indicate better performance in relation to IQ, Pattern Recognition Memory – Percent Correct, Rapid Visual Processing - Prime A Stockings of Cambridge – Minimum Moves, and worse performance in relation to all the remaining measures

**Table S6 - Bipolar I Disorder** - Within-twin/sibling cross-trait correlations, cross-twin/sibling within-trait correlations and cross-twin/sibling cross-trait correlations based on a constrained correlation (non-genetic) model ^a^

| **Cognitive measures ^b^** | ***Within-Twin/Sibling Cross-Trait Correlations*** | ***Cross-Twin/Sibling Within-Trait Correlations*** | | ***Cross-Twin/Sibling Cross-Trait Correlations*** | |
| --- | --- | --- | --- | --- | --- |
|  |  | ***Monozygotic (MZ)*** | ***Dizygotic (DZ)*** | ***Monozygotic (MZ)*** | ***Dizygotic (DZ)*** |
| IQ | -**0.14**  (-0.21/ -0.06) | **0.76**  (0.71/ 0.8) | **0.4**  (0.27/ 0.52) | -**0.15**  (-0.24/ -0.07) | 0.06  (-0.06/ 0.18) |
| Spatial Working Memory –  Between Errors | **0.17**  (0.09/ 0.25) | **0.53**  (0.42/ 0.62) | **0.37**  (0.23/ 0.49) | **0.11**  (0.02/ 0.21) | -0.03  (-0.15/ 0.1) |
| Spatial Working Memory –  Strategy | **0.11**  (0.03/ 0.19) | **0.37**  (0.24/ 0.49) | **0.32**  (0.18/ 0.45) | 0.1  (0/ 0.2) | -0.01  (-0.14/ 0.12) |
| Pattern Recognition Memory –  Mean Correct Latency | **0.11**  (0.02/ 0.2) | **0.45**  (0.31/ 0.56) | **0.49**  (0.27/ 0.64) | 0.02  (-0.08/ 0.12) | -0.08  (-0.21/ 0.05) |
| Pattern Recognition Memory –  Percent Correct | -0.08  (-0.18/ 0.01) | **0.56**  (0.43/ 0.66) | **0.35**  (0.04/ 0.55) | -0.1  (-0.2/ 0.01) | -0.01  (-0.15/ 0.13) |
| Rapid Visual Processing –  A Prime | -**0.16**  (-0.25/ -0.08) | **0.48**  (0.36/ 0.58) | **0.26**  (0.07/ 0.42) | -0.07  (-0.18/ 0.03) | -0.09  (-0.22/ 0.05) |
| Intra/Extra-Dimensional Shift –  Total Trials Adjusted | **0.12**  (0.03/ 0.21) | **0.27**  (0.13/ 0.4) | 0.09  (-0.09/ 0.26) | **0.13**  (0.02/ 0.24) | 0.01  (-0.13/ 0.16) |

^a^ Data were not available for Intra/Extra- Dimensional Shift- Total Errors Adjusted, Stockings of Cambridge- Minimum Moves, Stockings of Cambridge- Initial Thinking Time and Stockings of Cambridge- Subsequent Thinking Time.

^b^ Higher cognitive scores indicate better performance in relation to IQ, Pattern Recognition Memory – Percent Correct, Rapid Visual Processing - Prime A Stockings of Cambridge – Minimum Moves, and worse performance in relation to all the remaining measures

**Table S7** - **Broad Psychosis/Bipolar I Disorder Phenotype** - Within-twin/sibling cross-trait correlations, cross-twin/sibling within-trait correlations and cross-twin/sibling cross-trait correlations based on a constrained correlation (non-genetic) model

| **Cognitive measures ^a^** | ***Within-Twin/Sibling Cross-Trait Correlations*** | ***Cross-Twin/Sibling Within-Trait Correlations*** | | ***Cross-Twin/Sibling Cross-Trait Correlations*** | |
| --- | --- | --- | --- | --- | --- |
|  |  | ***Monozygotic (MZ)*** | ***Dizygotic (DZ)*** | ***Monozygotic (MZ)*** | ***Dizygotic (DZ)*** |
| IQ | -**0.24**  (-0.3/ -0.17) | **0.75**  (0.71/ 0.79) | **0.39**  (0.25/ 0.5) | **-0.2**  (-0.27/ -0.14) | -0.08  (-0.18/ 0.01) |
| Spatial Working Memory –  Between Errors | **0.24**  (0.17/ 0.31) | **0.52**  (0.41/ 0.61) | **0.36**  (0.22/ 0.49) | **0.2**  (0.12/ 0.28) | 0.07  (-0.03/ 0.17) |
| Spatial Working Memory –  Strategy | **0.13**  (0.06/ 0.2) | **0.37**  (0.24/ 0.49) | **0.32**  (0.18/ 0.45) | **0.13**  (0.05/ 0.21) | 0.04  (-0.07/ 0.14) |
| Pattern Recognition Memory –  Mean Correct Latency | **0.14**  (0.05/ 0.24) | **0.45**  (0.3/ 0.56) | **0.45**  (0.22/ 0.61) | 0.06  (-0.04/ 0.16) | -0.03  (-0.17/ 0.1) |
| Pattern Recognition Memory –  Percent Correct | -**0.17**  (-0.26/ -0.08) | **0.55**  (0.42/ 0.65) | **0.33**  (0.02/ 0.54) | -**0.18**  (-0.28/ -0.08) | -0.11  (-0.25/ 0.04) |
| Rapid Visual Processing –  A Prime | -**0.2**  (-0.27/ -0.12) | **0.47**  (0.35/ 0.57) | **0.26**  (0.07/ 0.42) | -**0.17**  (-0.25/ -0.09) | -0.07  (-0.18/ 0.04) |
| Intra/Extra-Dimensional Shift –  Total Trials Adjusted | **0.17**  (0.1/ 0.25) | **0.27**  (0.13/ 0.4) | 0.09  (-0.09/ 0.26) | **0.16**  (0.07/ 0.24) | 0.03  (-0.08/ 0.14) |
| Intra/Extra- Dimensional Shift–  Total Errors Adjusted | **0.22**  (0.12/ 0.31) | **0.33**  (0.15/ 0.49) | -0.21  (-0.44/ 0.09) | **0.2**  (0.1/ 0.31) | 0.08  (-0.07/ 0.23) |
| Stockings of Cambridge –  Minimum Moves | -**0.25**  (-0.35/ -0.14) | **0.45**  (0.28/ 0.59) | **0.48**  (0.26/ 0.63) | -**0.22**  (-0.33/ -0.11) | -**0.19**  (-0.33/ -0.04) |
| Stockings of Cambridge –  Initial Thinking Time | -0.09  (-0.20/ 0.01) | **0.34**  (0.15/ 0.50) | 0.10  (-0.14/ 0.31) | -0.06  (-0.17/ 0.05) | 0.01  (-0.14/ 0.16) |
| Stockings of Cambridge –  Subsequent Thinking Time | **0.21**  (0.09/ 0.32) | **0.62**  (0.46/ 0.72) | **0.34**  (0.15/ 0.5) | **0.24**  (0.12/ 0.36) | 0.09  (-0.06/ 0.24) |

^a^ Higher cognitive scores indicate better performance in relation to IQ, Pattern Recognition Memory- Percent Correct, Rapid Visual Processing- A Prime, Stockings of Cambridge- Minimum Moves, and worse performance in relation to all the remaining measures.

**Sensitivity Analysis**

**Table S8 - Schizophrenia** - Within-twin/sibling cross-trait correlations, cross-twin/sibling within-trait correlations and cross-twin/sibling cross-trait correlations based on a constrained correlation (non-genetic) model (Sensitivity Analysis)

| **Cognitive measures ^a^** | ***Within-Twin/Sibling Cross-Trait Correlations*** | ***Cross-Twin/Sibling Within-Trait Correlations*** | | ***Cross-Twin/Sibling Cross-Trait Correlations*** | |
| --- | --- | --- | --- | --- | --- |
|  |  | ***Monozygotic (MZ)*** | ***Dizygotic (DZ)*** | ***Monozygotic (MZ)*** | ***Dizygotic (DZ)*** |
| IQ | -**0.25**  (-0.32/ -0.17) | **0.76**  (0.71/ 0.8) | **0.39**  (0.25/ 0.5) | -**0.15**  (-0.22/ -0.07) | -0.11  (-0.22/ 0.01) |
| Spatial Working Memory –  Between Errors | **0.2**  (0.1/ 0.29) | **0.53**  (0.42/ 0.62) | **0.38**  (0.23/ 0.5) | **0.17**  (0.06/ 0.27) | 0  (-0.14/ 0.14) |
| Spatial Working Memory –  Strategy | 0.05  (-0.05/ 0.14) | **0.39**  (0.26/ 0.5) | **0.33**  (0.19/ 0.46) | **0.16**  (0.04/ 0.27) | -0.06  (-0.2/ 0.08) |
| Pattern Recognition Memory –  Mean Correct Latency | 0.13  (-0.01/ 0.26) | **0.44**  (0.3/ 0.56) | **0.45**  (0.21/ 0.61) | 0.06  (-0.09/ 0.21) | 0.01  (-0.25/ 0.26) |
| Pattern Recognition Memory –  Percent Correct | -**0.18**  (-0.31/ -0.05) | **0.56**  (0.43/ 0.66) | **0.35**  (0.05/ 0.55) | -**0.21**  (-0.36/ -0.06) | -0.14  (-0.4/ 0.14) |
| Rapid Visual Processing –  A Prime | -**0.13**  (-0.23/ -0.03) | **0.47**  (0.35/ 0.58) | **0.25**  (0.06/ 0.41) | -**0.14**  (-0.26/ -0.02) | 0.04  (-0.11/ 0.19) |
| Intra/Extra-Dimensional Shift –  Total Trials Adjusted | 0.09  (0/ 0.19) | **0.28**  (0.14/ 0.41) | 0.09  (-0.09/ 0.27) | 0.09  (-0.03/ 0.2) | -0.06  (-0.2/ 0.09) |
| Intra/Extra- Dimensional Shift–  Total Errors Adjusted | **0.14**  (0.04/ 0.24) | **0.34**  (0.15/ 0.49) | -0.18  (-0.43/ 0.11) | **0.16**  (0.03/ 0.28) | 0.02  (-0.13/ 0.18) |
| Stockings of Cambridge –  Minimum Moves | -**0.18**  (-0.29/ -0.07) | **0.46**  (0.28/ 0.6) | **0.48**  (0.27/ 0.63) | -**0.16**  (-0.29/ -0.03) | -0.13  (-0.27/ 0.03) |
| Stockings of Cambridge –  Initial Thinking Time | -0.11  (-0.21/ 0) | **0.35**  (0.15/ 0.51) | 0.1  (-0.13/ 0.31) | -0.01  (-0.13/ 0.11) | 0.04  (-0.12/ 0.2) |
| Stockings of Cambridge –  Subsequent Thinking Time | **0.12**  (0.01/ 0.23) | **0.62**  (0.47/ 0.73) | **0.35**  (0.06/ 0.51) | **0.19**  (0.06/ 0.31) | 0  (-0.16/ 0.15) |

^a^ Higher cognitive scores indicate better performance in relation to IQ, Pattern Recognition Memory- Percent Correct, Rapid Visual Processing- A Prime, Stockings of Cambridge- Minimum Moves, and worse performance in relation to all the remaining measures.

**Table S9 - Bipolar I Disorder** - Within-twin/sibling cross-trait correlations, cross-twin/sibling within-trait correlations and cross-twin/sibling cross-trait correlations based on a constrained correlation (non-genetic) model (Sensitivity Analysis) ^a^

| **Cognitive measures ^b^** | ***Within-Twin/Sibling Cross-Trait Correlations*** | ***Cross-Twin/Sibling Within-Trait Correlations*** | | ***Cross-Twin/Sibling Cross-Trait Correlations*** | |
| --- | --- | --- | --- | --- | --- |
|  |  | ***Monozygotic (MZ)*** | ***Dizygotic (DZ)*** | ***Monozygotic (MZ)*** | ***Dizygotic (DZ)*** |
| IQ | -**0.16**  (-0.24/ -0.07) | **0.76**  (0.71/ 0.80) | **0.4**  (0.26/ 0.52) | -**0.18**  (-0.27/ -0.08) | 0.06 (  -0.07/ 0.19) |
| Spatial Working Memory –  Between Errors | **0.19**  (0.1/ 0.28) | **0.53**  (0.42/ 0.62) | **0.37**  (0.23/ 0.49) | **0.12**  (0.01/ 0.23) | -0.02  (-0.16/ 0.12) |
| Spatial Working Memory –  Strategy | **0.12**  (0.03/ 0.21) | **0.37**  (0.24/ 0.49) | **0.32**  (0.18/ 0.45) | 0.11  (0/ 0.23) | -0.01  (-0.15/ 0.14) |
| Pattern Recognition Memory –  Mean Correct Latency | **0.12**  (0.02/ 0.22) | **0.45**  (0.31/ 0.56) | **0.49**  (0.27/ 0.63) | 0.01  (-0.11/ 0.13) | -0.09  (-0.23/ 0.06) |
| Pattern Recognition Memory –  Percent Correct | -0.09  (-0.2/ 0.01) | **0.56**  (0.43/ 0.66) | **0.34**  (0.04/ 0.55) | -0.11  (-0.23/ 0.01) | -0.02  (-0.18/ 0.14) |
| Rapid Visual Processing –  A Prime | -**0.19**  (-0.28/ -0.09) | **0.48**  (0.36/ 0.58) | **0.26**  (0.07/ 0.42) | -0.08  (-0.19/ 0.04) | -0.1  (-0.25/ 0.05) |
| Intra/Extra-Dimensional Shift –  Total Trials Adjusted | **0.14**  (0.04/ 0.24) | **0.27**  (0.13/ 0.4) | 0.09  (-0.09/ 0.26) | **0.15**  (0.02/ 0.27) | 0.02  (-0.14/ 0.18) |

^a^ Data were not available for Intra/Extra- Dimensional Shift- Total Errors Adjusted, Stockings of Cambridge- Minimum Moves, Stockings of Cambridge- Initial Thinking Time and Stockings of Cambridge- Subsequent Thinking Time.

^b^ Higher cognitive scores indicate better performance in relation to IQ, Pattern Recognition Memory – Percent Correct, Rapid Visual Processing - Prime A Stockings of Cambridge – Minimum Moves, and worse performance in relation to all the remaining measures.

**Table S10** – Additive genetic (*h*^2^), common environmental (*c*^2^) and unique environmental (*e*^2^) estimates for the neurocognitive measures based on the bivariate ACE genetic models for **Schizophrenia** and **Bipolar I Disorder** (sensitivity analysis) ^a^

| **Cognitive measures ^b^** | ***Schizophrenia*** | | | | | ***Bipolar I Disorder*** | | |
| --- | --- | --- | --- | --- | --- | --- | --- | --- |
|  | ***h^2^*** | ***c^2^*** | | ***e^2^*** | | ***h^2^*** | ***c^2^*** | ***e^2^*** |
| IQ | **0.73**  (0.51/ 0.8) | 0.03  (0/ 0.24) | | **0.24**  (0.2/ 0.29) | | **0.66**  (0.45/ 0.8) | 0.1  (0/ 0.3) | **0.24**  (0.2/ 0.29) |
| Spatial Working Memory –  Between Errors | **0.28**  (0.04/ 0.58) | 0.25  (0/ 0.47) | | **0.47**  (0.38/ 0.58) | | **0.29**  (0.02/ 0.59) | 0.24  (0/ 0.48) | **0.47**  (0.38/ 0.58) |
| Spatial Working Memory –  Strategy | Within-twin/sibling cross-trait correlation statistically non-significant – excluded from further analysis | | | | | 0.08  (0/ 0.43) | 0.29  (0/ 0.42) | **0.63**  (0.51/ 0.74) |
| Pattern Recognition Memory –  Mean Correct Latency | Within-twin/sibling cross-trait correlation statistically non-significant – excluded from further analysis | | | | | 0  (0/ 0.03) | **0.46**  (0.11/ 0.56) | **0.54**  (0.44/ 0.63) |
| Pattern Recognition Memory –  Percent Correct | **0.41**  (0.01/ 0.66) | | 0.14  (NA/ 0.54) | | **0.44**  (0.34/ 0.57) | Within-twin/sibling cross-trait correlation statistically non-significant – excluded from further analysis | | |
| Rapid Visual Processing –  A Prime | **0.36**  (0.03/ 0.57) | 0.12  (0/ 0.4) | | **0.53**  (0.43/ 0.61) | | **0.43**  (0.09/ 0.58) | 0.05  (0/ 0.37) | **0.52**  (0.42/ 0.64) |
| Intra/Extra-Dimensional Shift –  Total Trials Adjusted | Within-twin/sibling cross-trait correlation statistically non-significant – excluded from further analysis | | | | | **0.26**  (0.01/ 0.38) | 0  (0/ 0.25) | **0.74**  (0.62/ 0.88) |
| Intra/Extra- Dimensional Shift–  Total Errors Adjusted | **0.27**  (0.01/ 0.45) | | 0  (0/ 0.22) | | **0.73**  (0.55/ 0.92) | Data not available | | |
| Stockings of Cambridge –  Minimum Moves | 0.01  (0/ 0.45) | | **0.46**  (0.27/ 0.58) | | **0.53**  (0.42/ 0.66) | Data not available | | |
| Stockings of Cambridge –  Initial Thinking Time | Within-twin/sibling cross-trait correlation statistically non-significant – excluded from further analysis | | | | | Data not available | | |
| Stockings of Cambridge –  Subsequent Thinking Time | **0.49**  (0.11/ 0.72) | | 0.13  (0/ 0.44) | | **0.38**  (0.27. 0.53) | Data not available | | |

^a^ *h*^2^, *c*^2^ and *e*^2^ indicate heritability, shared environmental and unique environmental effects, respectively. Results in bold have a statistically significant point estimate (the 95% confidence interval excludes 0). Only results for neurocognitive measures that showed statistically significant within-twin/sibling cross-trait correlations with the diagnostic phenotype are reported.
^b^ Higher cognitive scores indicate better performance in relation to IQ, Pattern Recognition Memory – Percent Correct, Rapid Visual Processing - Prime A Stockings of Cambridge – Minimum Moves, and worse performance in relation to all the remaining measures.

**Table S11 - Schizophrenia** - Additive genetic (*h*^2^) estimates, phenotypic correlations with neurocognitive measures, decomposed sources of correlations based on the bivariate ACE models, and A, C and E correlation estimates ^a^ (Sensitivity Analysis)

| **Cognitive measures ^b^** | ***h^2^*** | ***r*_ph-a_** | ***r*_ph-c_** | ***r*_ph-e_** | ***r*_ph_** | ***r*_g_** | ***r*_c_** | ***r*_e_** |
| --- | --- | --- | --- | --- | --- | --- | --- | --- |
| IQ | **0.73**  (0.51/ 0.8) | -**0.13**  (-0.24/ -0.03) | -0.02  (-0.06/ 0.05) | -**0.1**  (-0.14/ -0.05) | -**0.24**  (-0.31/ -0.17) | -**0.17**  (-0.34/ -0.05) | -1  (NA/ 1) | -**0.43**  (-0.62/ -0.21) |
| Spatial Working Memory –  Between Errors | **0.28**  (0.04/ 0.58) | **0.22**  (0.11/ 0.29) | -0.06  (-0.08/ 0.07) | 0.04  (-0.04/ 0.11) | **0.2**  (0.11/ 0.29) | **0.48**  (0.19/ 1) | -1  (NA/ 1) | 0.12  (-0.12/ 0.35) |
| Spatial Working Memory –  Strategy | Within-twin/sibling cross-trait correlation statistically non-significant – excluded from further analysis | | | | | | | |
| Pattern Recognition Memory –  Mean Correct Latency | Within-twin/sibling cross-trait correlation statistically non-significant – excluded from further analysis | | | | | | | |
| Pattern Recognition Memory –  Percent Correct | **0.41**  (0.01/ 0.66) | **-0.17**  (-0.41/ -0.01) | -0.04  (-0.08/ 0.08) | 0.03  (-0.13/ 0.17) | -**0.18**  (-0.31/ -0.05) | -**0.3**  (-1/ -0.3) | -1  (NA/ 1) | 0.1  (-0.41/ 0.56) |
| Rapid Visual Processing –  A Prime | **0.36**  (0.03/ 0.57) | -0.17  (-0.29/ 0.01) | 0.04  (-0.06/ 0.07) | -0.01  (-0.11/ 0.09) | -**0.14**  (-0.24/ -0.04) | -0.32  (-1/ 0.02) | 1  (-1/ 1) | -0.03  (-0.32/ 0.26) |
| Intra/Extra-Dimensional Shift –  Total Trials Adjusted | Within-twin/sibling cross-trait correlation statistically non-significant – excluded from further analysis | | | | | | | |
| Intra/Extra- Dimensional Shift–  Total Errors Adjusted | **0.27**  (0.01/ 0.45) | **0.15**  (0.01/ 0.29) | 0  (-0.05/ 0.05) | 0  (-0.12/ 0.11) | **0.15**  (0.04/ 0.25) | **0.33**  (0.03/ 1) | -1  (NA/ 1) | -0.01  (-0.29/ 0.27) |
| Stockings of Cambridge –  Minimum Moves | 0.01  (0/ 0.45) | -0.08  (-0.21/ 0.05) | -**0.08**  (-0.09/ -0.06) | -0.02  (-0.12/ 0.08) | -**0.18**  (-0.28/ -0.08) | -1  (-1/ 1) | -1  (NA/ 1) | -0.06  (-0.36/ 0.24) |
| Stockings of Cambridge –  Initial Thinking Time | Within-twin/sibling cross-trait correlation statistically non-significant – excluded from further analysis | | | | | | | |
| Stockings of Cambridge –  Subsequent Thinking Time | **0.49**  (0.11/ 0.72) | **0.23**  (0.04/ 0.36) | -0.04  (-0.08/ 0.07) | -0.05  (-0.14/ 0.04) | **0.13**  (0.02/ 0.24) | **0.37**  (0.07/ 0.99) | -1  (-1/ 1) | -0.18  (-0.47/ 0.13) |

^a^ *h*^2^ indicates heritability. Results in bold have a statistically significant point estimate (the 95% confidence interval excludes 0). Only results for neurocognitive measures that showed statistically significant within-twin/sibling cross-trait correlations with the diagnostic phenotype are reported. The a and g subscripts in ***r*_ph-a_** and ***r*_g_** both refer to additive genetic effects.

^b^ Higher cognitive scores indicate better performance in relation to IQ Pattern Recognition Memory – Percent Correct, Rapid Visual Processing - Prime A Stockings of Cambridge – Minimum Moves, and worse performance in relation to all the remaining measures.

† The measure satisfied all criteria for endophenotypes.

**Table S12 - Bipolar I Disorder** - Additive genetic (*h*^2^) estimates, phenotypic correlations with neurocognitive measures, decomposed sources of correlations based on the bivariate ACE models, and A, C and E correlation estimates ^a^ (Sensitivity Analysis)

| **Cognitive measures ^b^** | ***h^2^*** | ***r*_ph-a_** | ***r*_ph-c_** | ***r*_ph-e_** | ***r*_ph_** | ***r*_g_** | ***r*_c_** | ***r*_e_** |
| --- | --- | --- | --- | --- | --- | --- | --- | --- |
| IQ | **0.66**  (0.45/ 0.8) | -**0.19**  (-0.28/ -0.07) | 0.02  (-0.03/ 0.03) | 0  (-0.06/ 0.06) | -**0.16**  (-0.25/ -0.08) | -**0.26**  (-0.43/ -0.09) | 1  (-1/ NA) | 0.01  (-0.24/ 0.27) |
| Spatial Working Memory –  Between Errors | **0.29**  (0.02/ 0.59) | **0.14**  (0.04/ 0.25) | -0.03  (-0.04/ 0.04) | 0.08  (-0.01/ 0.17) | **0.19**  (0.1/ 0.28) | **0.31**  (0.07/ 1) | -1  (NA/ 1) | 0.24  (-0.03/ 0.49) |
| Spatial Working Memory –  Strategy | 0.08  (0/ 0.43) | **0.14**  (0.02/ 0.25) | -0.03  (-0.04/ 0.04) | 0.02  (-0.08/ 0.13) | **0.12**  (0.03/ 0.21) | **0.55**  (0.55/ 1) | -1  (-1/ 1) | 0.06  (-0.22/ 0.33) |
| Pattern Recognition Memory –  Mean Correct Latency | 0  (0/ 0.03) | 0.05  (-0.07/ 0.16) | -**0.04**  (-0.05/ -0.04) | **0.13**  (0.04/ 0.22) | **0.14**  (0.04/ 0.23) | 1  (-1/ NA) | -1  (-1/ 1) | **0.37**  (0.11/ 0.6) |
| Pattern Recognition Memory –  Percent Correct | Within-twin/sibling cross-trait correlation statistically non-significant – excluded from further analysis | | | | | | | |
| Rapid Visual Processing –  A Prime | **0.43**  (0.09/ 0.58) | -0.07  (-0.22/ 0.05) | -0.01  (-0.04/ 0.04) | -0.1  (-0.2/ 0.01) | -**0.19**  (-0.28/ -0.09) | -0.13  (-0.3/ 0.11) | -1  (-1/ 1) | -0.28  (-0.56/ 0.03) |
| Intra/Extra-Dimensional Shift –  Total Trials Adjusted | **0.26**  (0.01/ 0.38) | 0.14  (0/ 0.27) | 0  (-0.03/ 0.03) | 0.01  (-0.12/ 0.13) | **0.14**  (0.04/ 0.24) | **0.32**  (0.32/ 1) | -1  (-1/ 1) | 0.01  (-0.29/ 0.31) |

^a^ *h*^2^ indicates heritability. Results in bold have a statistically significant point estimate (the 95% confidence interval excludes 0). Only results for neurocognitive measures that showed statistically significant within-twin/sibling cross-trait correlations with the diagnostic phenotype are reported. The a and g subscripts in ***r*_ph-a_** and ***r*_g_** both refer to additive genetic effects. Data were not available for Intra/Extra- Dimensional Shift- Total Errors Adjusted, Stockings of Cambridge- Minimum Moves, Stockings of Cambridge- Initial Thinking Time and Stockings of Cambridge- Subsequent Thinking Time.

^b^ Higher cognitive scores indicate better performance in relation to IQ, Pattern Recognition Memory – Percent Correct, Rapid Visual Processing - Prime A Stockings of Cambridge – Minimum Moves, and worse performance in relation to all the remaining measures.

† The measure satisfied all criteria for endophenotypes.

**Table S13** Mean (SD) neurocognitive scores in participants with schizophrenia, bipolar I disorder with a positive lifetime history of psychotic symptoms, bipolar I disorder with a negative lifetime history of psychotic symptoms, and unaffected by psychotic/bipolar disorders

|  |  | **Schizophrenia (n=145)** |  | **Bipolar I Disorder (n=73) ^a^** | | |  | **Unaffected (n=793)** |  | **Statistical comparison ^b^** |
| --- | --- | --- | --- | --- | --- | --- | --- | --- | --- | --- |
|  |  |  |  | Lifetime history (+) of psychotic symptoms  (n=56) |  | No lifetime history (-) of psychotic symptoms  (n=17) |  |  |  |  |
|  |  |  |  |  |  |  |  |  |  |  |
|  |  | (Group 1) |  | (Group 2) |  | (Group 3) |  | (Group 4) |  |  |
|  |  |  |  |  |  |  |  |  |  |  |
| **Cognitive measures ^c^** |  | Mean (SD) |  | Mean (SD) |  | Mean (SD) |  | Mean (SD) |  |  |
|  |  |  |  |  |  |  |  |  |  |  |
| IQ |  | 94.06 (14.23) |  | 110.66 (12.20) |  | 105.82 (12.62) |  | 108.95 (13.74) |  | *P*<0.001 ^d^ |
| Spatial Working Memory – Between Errors |  | 36.34 (26.80) |  | 29.93 (21.23) |  | 37.07 (19.14) |  | 21.26 (17.36) |  | *P*<0.001 ^e^ |
| Spatial Working Memory - Strategy |  | 32.57 (7.46) |  | 32.96 (5.52) |  | 34.80 (4.77) |  | 30.92 (6.40) |  | *P*<0.01 ^f^ |
| Pattern Recognition Memory – Mean Correct Latency |  | 2572.63 (1016.43) |  | 2268.42 (670.46) |  | 2197.35 (306.36) |  | 2056.92 (523.94) |  | *P*<0.01 ^g^ |
| Pattern Recognition Memory - Percent Correct |  | 74.87 (19.68) |  | 88.73 (11.46) |  | 86.94 (6.07) |  | 90.99 (8.47) |  | *P*<0.001 ^h^ |
| 3Rapid Visual Processing – A Prime |  | 0.87 (0.07) |  | 0.89 (0.05) |  | 0.88 (0.05) |  | 0.91 (0.05) |  | *P*<0.001 ^i^ |
| Intra/Extra Dimensional Shift – Total Trials Adjusted |  | 143.81 (95.96) |  | 95.55 (30.54) |  | 94.33 (29.91) |  | 92.65 (42.22) |  | *P*<0.001 ^j^ |
|  |  |  |  |  |  |  |  |  |  |  |

^a^ Of the extended sample of participants with bipolar I disorder (n=86), 13 did not have available information on ‘lifetime history of psychotic symptoms’.
^b^ Statistical group comparisons in neurocognitive scores were performed using linear regression analysis with robust standard errors and with the cluster option to account for the dependency of observations within twin or sibling pairs (clusters). The degrees of freedom in these models refer to the number of clusters rather than participants. Statistical results with P values < 0.007 survive correction for multiple comparisons (Statistical threshold of 0.05 divided by 7 cognitive variables = 0.007).
^c^ Higher cognitive scores indicate better performance in relation to IQ, Pattern Recognition Memory – Percent Correct, and Rapid Visual Processing – A Prime, and worse performance in relation to the remaining measures. Data are not presented for Intra/Extra-Dimensional Shift-Total Errors Adjusted and for Stockings of Cambridge variables, as these were not available in the bipolar I disorder group.
^d^ F(1, 485)=59.57, *P*<0.001: Group 1 < Group 2, Group 3, Group 4
^e^ F(1, 381)=29.23, *P*<0.001: Group 1, Group 2, Group 3 > Group 4
^f^ F(1, 381)=7.11, *P*<0.01: Group 2, Group 3 > Group 4
^g^ F(1, 212)=9.40, *P*<0.01: Group 1, Group 2 > Group 4
^h^ F(1, 212)=14.41, *P*<0.001: Group 1 < Group 2, Group 3, Group 4; and Group 3 < Group 4
^i^ F(1, 335)=24.88, *P*<0.001: Group 1, Group 2, Group 3 < Group 4
^j^ F(1, 336)=14.67, *P*<0.001: Group 1 > Group 2, Group 3, Group 4

**Supplementary Figures**


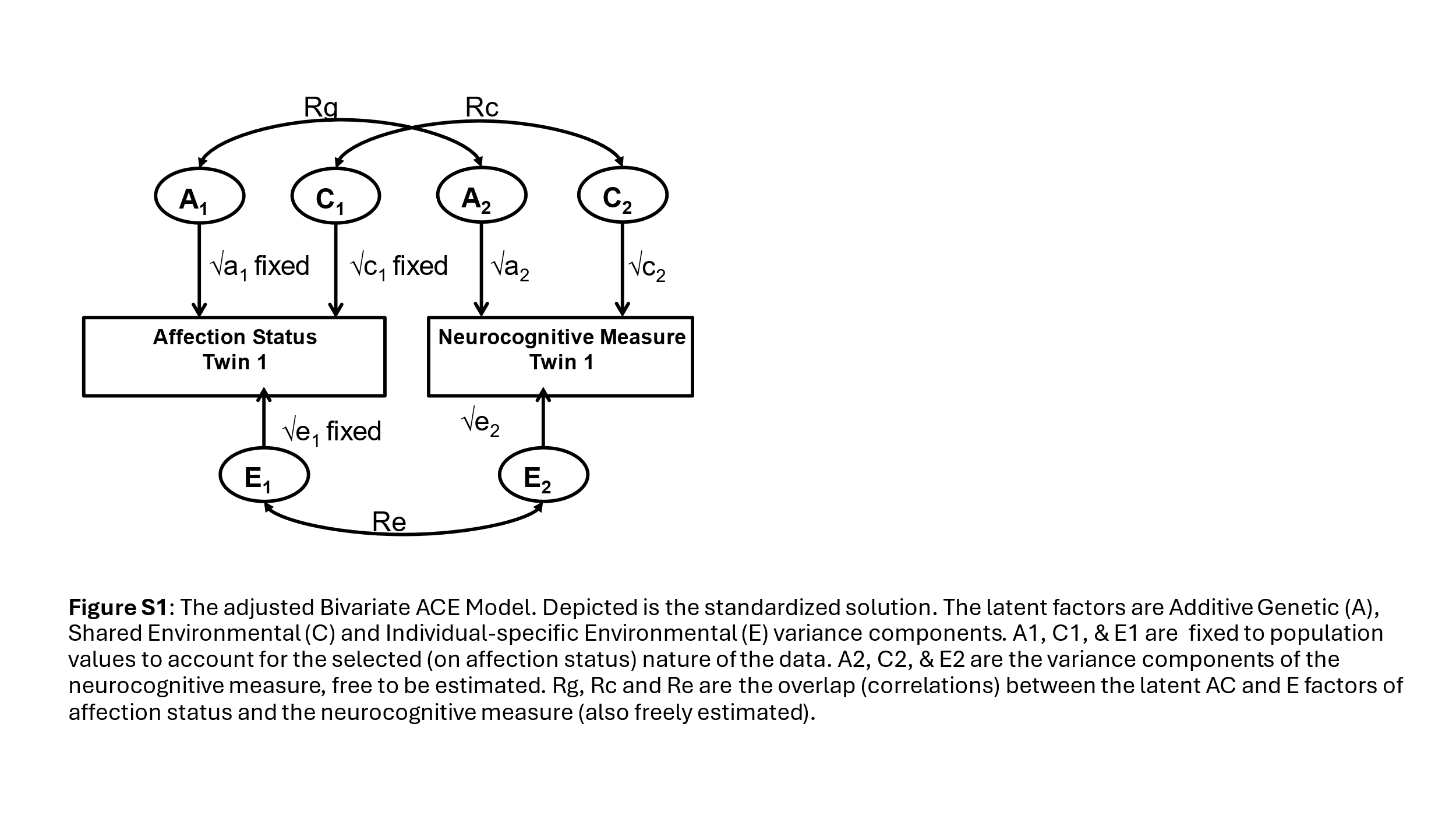


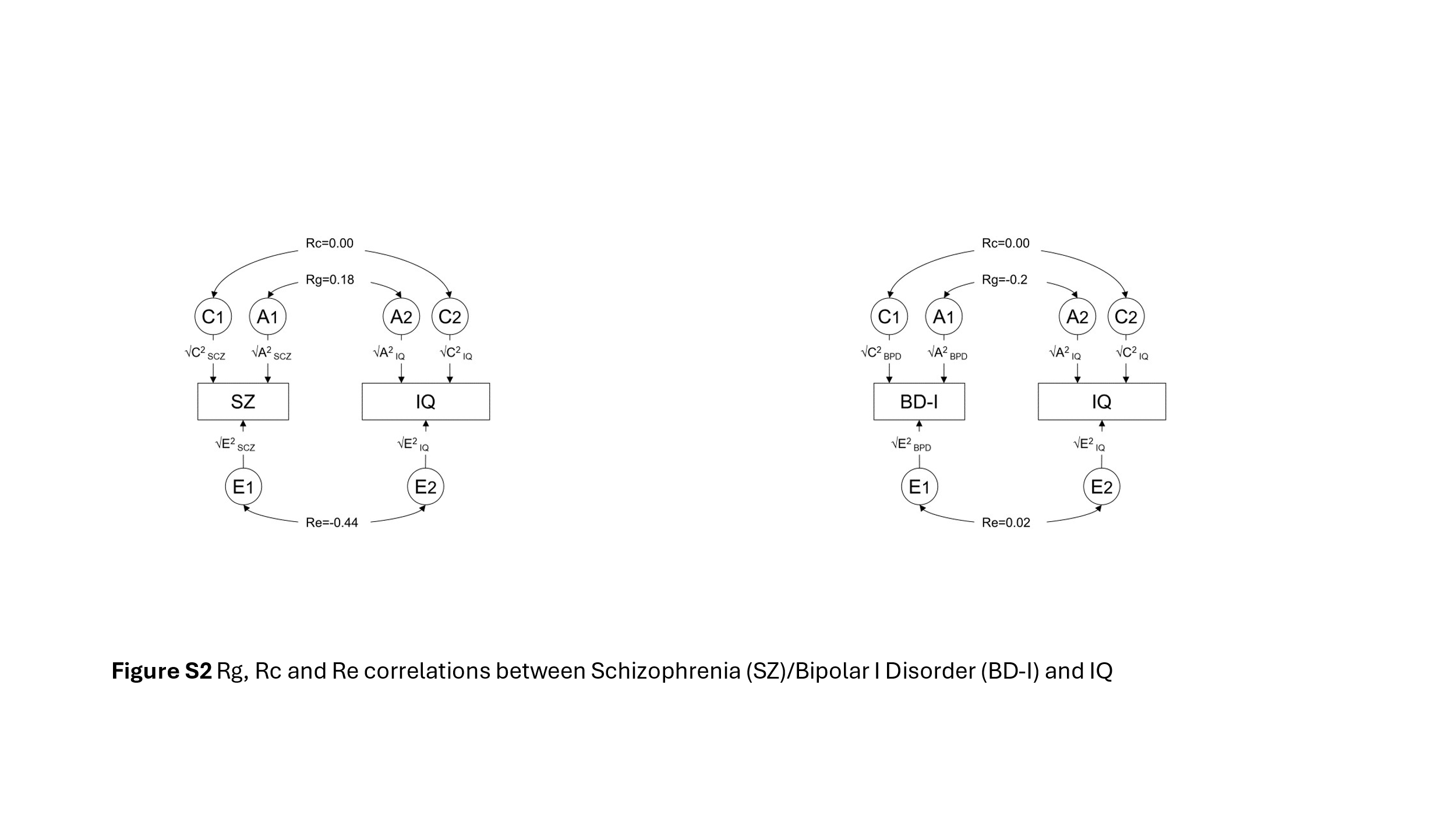


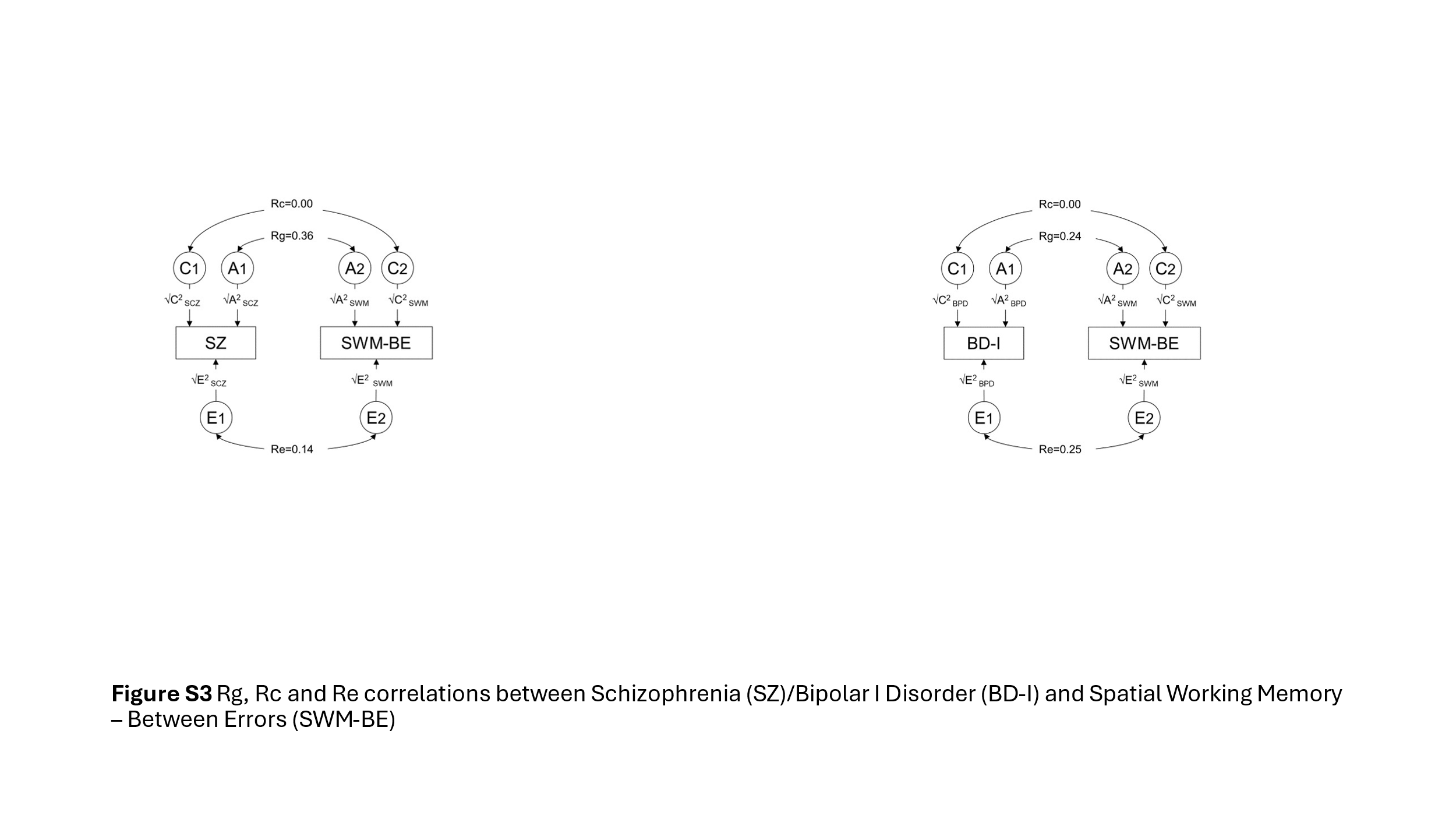


**Figure S4** Genetic correlations (*r*_g_)^a^ between diagnostic phenotypes and neurocognitive measures


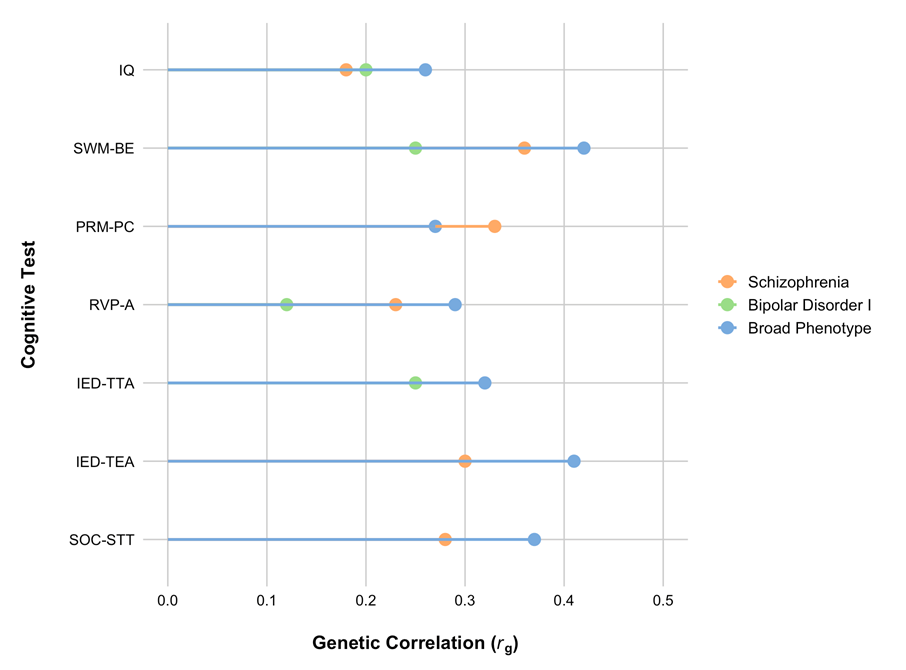


Abbreviations: **IED-TTA:** Intra- Extra Dimensional Set Shift Total Trials Adjusted; **IED-TEA**: Intra- Extra Dimensional Set Shift Total Errors Adjusted; **PRM- PC:** Pattern Recognition Memory Percent Correct; **RVP-A**: Rapid Visual Information Processing signal detection measure A Prime; **SOC-STT**: Stockings of Cambridge Mean Subsequent Thinking Time (highest difficulty level); **SWM- BE**: Spatial Working Memory Between Errors

^a^ Genetic correlations (*r*_g_) are presented only for measures with heritabilities (*h*^2^)>10%. Of the results presented, only IQ and SWM-BE showed statistically significant genetic correlations (*r*_g_) with all three diagnostic phenotypes.

**Appendix A -** Detailed Description of the Analytic Cohort

The analytic cohort comprised 1050 participants drawn from twin and sibling samples described in earlier studies (Toulopoulou et al., 2007; Owens et al., 2011a; Owens et al., 2011b; Owens et al, 2012; Georgiades et al., 2016; Lemvigh et al., 2020; Bootsman et al., 2015). Only participants aged 16-65 years with IQ=>70 were included in the present analysis. This criterion led to an exclusion of 31 participants (18%) from the original UK and Danish schizophrenia cohorts. The analytic cohort included 145 (108 MZ) twins with schizophrenia, 75 (52 MZ) twins and 11 siblings with bipolar I disorder (BD-I), 26 (16 MZ) twins with other psychotic disorders (schizoaffective disorder, depressive psychosis, schizotypal disorder, brief psychotic disorder, unspecified non-organic psychosis), and 735 (438 MZ) twins and 58 siblings unaffected by psychotic or bipolar disorders. The latter were drawn from 287/24 complete control twin/sibling pairs with no personal or family histories of psychotic or bipolar disorders; from 15 incomplete control pairs; from 120/9 complete twin/sibling pairs discordant for schizophrenia, bipolar I or other psychotic disorders; and from 26/1 incomplete twin/sibling pairs discordant for SZ, BD-I or other psychotic disorders.

The schizophrenia cohort comprised 70 MZ twins from 32 complete and six incomplete twin pairs concordant for schizophrenia, 71 twins (36 MZ) from 66 complete and five incomplete twin pairs discordant for schizophrenia, and 4 (2 MZ) twins with SZ whose participating co-twins had non-schizophrenic, non-bipolar psychotic disorders.

The bipolar I disorder cohort comprised 29 (25 MZ) twins from 13 complete and three incomplete twin pairs concordant for bipolar I disorder; 37 (23 MZ) twins and 11 siblings from 36/1 complete/incomplete twin pairs and from 9/2 complete/incomplete sibling pairs discordant for bipolar I disorder; seven (4 MZ) twins with BD-I whose non-bipolar co-twins had a psychotic disorder other than schizophrenia; and two DZ BD-I twins whose non-participating co-twins had unspecified diagnostic status. Of the 86 BD-I participants, 56 (65.12%) had a recorded history of psychotic symptoms.

The sample of other psychotic disorders comprised eight (5 MZ) twins with brief psychotic disorders from 7/1 complete/incomplete twin pairs, whose co-twins were unaffected by psychotic or bipolar disorders; six (3 MZ) twins with schizoaffective disorder whose co-twins included two BD-I participants and four participants unaffected by psychotic or bipolar disorders; two (1 MZ) twins with depressive psychosis whose participating co-twins had BD-I; nine (7 MZ) twins with schizotypal disorder whose co-twins included six participants unaffected by psychotic disorders, one participating co-twin with schizophrenia, one participating co-twin with autism spectrum disorder, and one non-participating co-twin with a psychotic disorder; and a DZ twin with unspecified non-organic psychosis whose non-participating co-twin was unaffected by psychotic or bipolar disorders.

The analytic cohort finally included 735 (438 MZ) twins and 58 siblings unaffected by psychotic or bipolar disorders. These were drawn from 287/24 complete control twin/sibling pairs with no personal or family histories of psychotic or bipolar disorders; from 15 incomplete control pairs; from 120/9 complete twin/sibling pairs discordant for schizophrenia, bipolar I or other psychotic disorders; and from 26/1 incomplete twin/sibling pairs discordant for SZ, BD-I or other psychotic disorders.

Non-psychotic, non-bipolar psychiatric pathology was not an exclusion criterion for any of the study groups in the present analysis. Of the 793 unaffected twins and siblings, 82 had personal histories of unipolar depressive disorders (n=46), neurotic, stress-related and somatoform disorders (n=15), mental and behavioural disorders due to psychoactive substance use (n=7), autism-spectrum disorders (n=1), disorders of adult personality and behaviour (n=1), or a combination of the aforementioned diagnoses (n=12). No participant was acutely ill at the time of data collection.

With the exception of 14 twin pairs, all co-twins/co-siblings were of the same sex. The mean (SD) age difference between co-siblings was 3.6 (3.0) years.

**Appendix B -** Description and Rationale for the Sample Structure of the Mega-analysis

Three separate model-fitting analyses were performed, one for each diagnostic phenotype:

- **Analysis I - Schizophrenia (SZ):** Affected (narrow SZ phenotype=145 participants) versus Unaffected (all non-SZ, non-psychotic, non-bipolar participants from unaffected and discordant twin/sibling pairs=793 participants)
- **Analysis II - Bipolar I Disorder (BD-I):** Affected (narrow BD-I phenotype=86 participants) versus Unaffected (all non-SZ, non-psychotic, non-bipolar participants from unaffected and discordant twin/sibling pairs=793 participants)
- **Analysis III - Broad Psychosis/BD-I phenotype:** Affected (narrow SZ phenotype/145 participants + narrow BD-I phenotype/86 participants + other primary psychoses/26 participants=257 participants) versus Unaffected (all non-SZ, non-psychotic, non-bipolar participants from unaffected and discordant twin/sibling pairs=793 participants)

We defined affection status relatively narrowly for analysis I and II, limiting to a diagnosis of SZ and of BD-I respectively, including to potentially highlight contrasting associations for these diagnoses. For analysis III, we defined a broad psychosis/BD-I phenotype, including to potentially highlight associations shared in common across these disorders which have partial overlap in risk factors. For each analysis, we defined unaffected status as having none of the disorders included in the affected status definitions in order to achieve optimal contrast between affected and unaffected status.

The model-fitting method we used does not infer a genetic link by comparing means, but estimates the phenotypic correlation between two variables: the liability to the disorder (0/1) and the score on the psychometric variable by using a mixed ordinal-continuous maximum likelihood modelling approach. In addition, it is the differential correlation pattern observed in the MZ and DZ pairs (cross-trait within twins, cross-trait cross-twins) that provides the statistical power to decompose this phenotypic correlation into a genetic and environmental component. In summary, these analyses use the following information for each individual:

- Are they above or below the threshold for the disorder? (affection status yes/no)
- What is their score on the endophenotype?
- Are they a member of an MZ/DZ/sibling pair?

The important covariates (age, gender, education, research centre) are all regressed out in the means of the psychometric variables. This has the same effect as ‘matching’ groups in terms of covariates.
